# Exploring the bidirectional causality between neuroticism and frailty: a Mendelian randomization analysis

**DOI:** 10.1186/s41065-025-00370-2

**Published:** 2025-01-25

**Authors:** Yuhang Xing, Rui Pu, Mengdie Fu, Zhikang Wang, Zhen Wang, Xiaopeng Shang, Guoli Yang, Zhiwei Jiang

**Affiliations:** 1 Zhejiang Provincial Center for Disease Control and Prevention, Hangzhou, China; 2 Stomatology Hospital, School of Stomatology, Zhejiang University School of Medicine, Zhejiang Provincial Clinical Research Center for Oral Diseases, Key Laboratory of Oral Biomedical Research of Zhejiang Province, Cancer Center of Zhejiang University, Engineering Research Center of Oral Biomaterials and Devices of Zhejiang Province, Hangzhou, China

**Keywords:** Frailty, Mental disorder, Neuroticism, Mendelian randomization

## Abstract

**Background:**

Epidemiological studies have confirmed the relationship between personality trait neuroticism and physical health. However, the relationship between neuroticism and frailty remains unconfirmed. This study employed a bi-directional two-sample Mendelian randomization (MR) approach to investigate the causal relationship between neuroticism and frailty.

**Methods:**

The neuroticism genome-wide association study (GWAS) data from the UK Biobank contained twelve neuroticism-related traits with 489,212 participants. The genetic frailty index data were extracted from the UK Biobank and Swedish TwinGene, involving 175,226 individuals. Independent genetic variants associated with neuroticism and frailty were selected as instrumental variables. Inverse variance weighted (IVW), MR-Egger, weighted median, weighted mode, and MR-PRESSO were mainly used for MR analysis.

**Results:**

The MR analysis showed a positive causal relationship between neuroticism and the risk of frailty (odds ratio (OR) = 1.627, 95% confidence interval (CI) = 1.538–1.722, *P* < 0.001). In the reverse direction, frailty had a causal effect on a higher risk of neuroticism (OR = 1.270, 95% CI = 1.173–1.375, *P* < 0.001). Steiger tests indicated that reverse causation did not bias the identified causal relationships.

**Conclusions:**

Our study provides genetic evidence suggesting a bi-directional causal relationship between frailty and neuroticism. In this bi-directional MR study, there were positive causal relationships between neuroticism-related phenotypes and frailty, and in the reverse direction, frailty was also positively correlated with neuroticism.

**Supplementary Information:**

The online version contains supplementary material available at 10.1186/s41065-025-00370-2.

## Introduction

Frailty is a significant public health challenge worldwide [1, 2]. Rockwood et al. described frailty as a complex, multi-dimensional condition characterized by the loss of various reserves such as wealth, physical strength, intellect, and health, thereby increasing an individual’s vulnerability [3]. The high prevalence of frailty places a serious burden on older adults, families, and society. However, there are no specific medications to prevent or treat frailty. Non-pharmacological interventions remain one of the primary means of preventing and treating frailty, such as nutritional interventions or placebo [4]. Frailty is commonly measured using the frailty index (FI), which is based on accumulating numerous health deficits throughout the life course [5]. Higher FI levels are associated with many adverse health outcomes, including disability, reduced mobility, a range of chronic diseases and hospitalizations, and mortality [6, 7]. Less is known about how psychological factors might be contributors to frailty.

Neuroticism is the propensity to experience negative emotions, including anxiety, fear, sadness, anger, guilt, disgust, irritability, loneliness, worry, self-consciousness, dissatisfaction, hostility, embarrassment, reduced self-confidence, and feelings of vulnerability, in reaction to various types of stress [8]. This trait also predisposes individuals to various mental health disorders, including anxiety, mood, substance, somatic, and eating [9, 10]. Moreover, neuroticism is associated with physical conditions such as heart problems, impaired immune function, asthma, atopic eczema, irritable bowel syndrome, and even an increased risk of mortality [11].

Previous studies have identified several sociodemographic and health-related determinants of frailty [12, 13]. A cohort study involving 10,317 people aged over 65 years found high neuroticism was further related to a steeper worsening of frailty [14]. Five cohorts or cross-sectional data revealed that higher neuroticism had a causal influence on frailty [15]. While McHugh et al. reported that neuroticism appears to have no causal impact on frailty transitions in older adults after two years of follow-up [16]. A cohort of 4,339 individuals found that increased frailty was not associated with neuroticism [17]. Thus, the precise causal relationship between frailty and neuroticism remains unclear.

Mendelian randomization (MR) is a method of causal inference based on genetic variants; genetic variation associated with exposure to the research subject is used as an instrumental variable [18]. Genetic variation is assigned by combining meiosis, producing a random distribution of genetic variation in the population, and the genetic associations observed in MR analyses are not subject to confounding bias and reverse causation risk [18, 19]. Thus, MR is considered a complementary approach to randomized controlled trials, providing a reliable understanding of the effects of modifiable exposures on features of interest [20]. Given the lack of solid evidence from observational results, MR may be a useful complementary tool to explore the causal relationship between neuroticism and frailty. Therefore, this study aimed to assess the relationship between neuroticism and frailty. It was evaluated by MR analysis using publicly available genetic data.

## Methods

### Study design

**Fig. 1:**
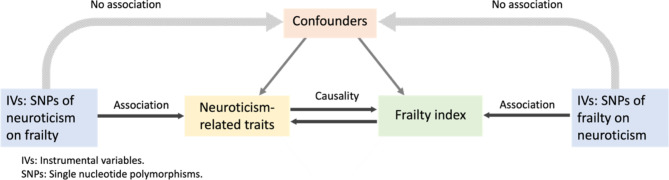
The bidirectional Mendelian randomization concept framework. IV, instrumental variables; SNP, single nucleotide polymorphism

We developed a bidirectional MR approach, as shown in Fig. 1. For this MR study, we applied multiple two-sample analyses and relied on openly accessible summary statistics based on extensive GWAS datasets. MR’s central idea is that a genetic variation’s genotype determines a different intermediate phenotype. If the phenotype is a particular exposure characteristic of an individual, the association assessment of genotype and disease should be able to simulate the effect of exposure factors on the disease.

### Data sources

The UK Biobank Study is a large data resource, containing phenotypic measures from 503,325 participants and genetic data from 489,212 participants [21]. Neuroticism was measured using the Eysenck Personality Questionnaire, Revised Short Form (EPQ-R-S) [22], which consists of 12 dichotomous items (‘yes’ or ‘no’). Participants who completed < 9 items were excluded from further analysis. Neuroticism trait samples were stained with 12 dichotomous neuroticism including neuroticism, irritable mood, feeling lonely, feeling miserable, experiencing mood swings, feeling guilty, worry too long after an embarrassing experience, feeling fed-up, feeling nervous, feeling worry, feeling hurt, feeling tense [23]. Individuals were excluded from analyses if they had missing responses to more than 3 (out of 12) neuroticism items. At the time of completion of the test, the ages of the participants ranged from 40 to 73 years (Mean ± SD: 56.91 ± 7.93). The summary statistics of the GWAS for each neuroticism trait are publicly available in the GWAS Catalog (accession numbers ranging from GCST006940 to GCST006948, GCST006950 to GCST006952).

The genetic data of the FI were extracted from the Integrative Epidemiology Unit (IEU) open GWAS project (ebi-a-GCST90020053). It was obtained from UK BioBank which comprised a total of 175,226 subjects and 7,589,717 single nucleotide polymorphisms (SNPs) with European ancestry. The FI was calculated using 49 self-reported symptoms, disability, and diagnosed disease items for UK Biobank (*N* = 164,610, 60 to 70 years) and Swedish TwinGene (*N* = 10,616, 41 to 87 years), respectively [24].

### Instrumental variable selection

Instrumental variable (IV) is used to solve endogenous problems, such as confounding factors, measurement bias, and temporal confusion. Factors that can be used as IV must meet three conditions: (1) Correlation: IV and exposure factors must have a robust and strong correlation; (2) Independence: IV must be independent of confounding factors; (3) Exclusivity: IV must influence outcomes only through exposure factors.

The study rigorously selects significant IVs to reduce false positives. It also reduces the bias of linkage disequilibrium. IVs were chosen based on a significance level of *P* < 5 × 10^− 8^. The selected SNPs were clustered to realize an independent inheritance, with linkage disequilibrium r^2^ < 0.001 parameters and distance window 10,000 kb [25]. We calculated the proportion of phenotypic variation explained (PVE) and then used the instrumental F statistics for each immune trait to assess the strength of the IVs and avoid weak instrumental bias in the MR analysis. IVs with low F-statistics (< 10) were removed from the analysis.

The PVE and F-statistic were calculated using the formula below:

$$ {\rm{PVE}}\,{\rm{ = }}\,{\rm{2}}\,{\rm{ \times }}\,{\rm{EAF}}\,{\rm{ \times }}\,\left( {{\rm{1 - EAF}}} \right)\,{\rm{ \times }}\,{{\rm{\beta }}^{\rm{2}}} $$

(EAF, effect allele frequency; β, effect size on the exposure)

$${\rm{F - statistic}}\, = \frac{{{\rm{PVE}}\: \times \:\:({\rm{n}}\: - \:1\: - \:{\rm{k}})}}{{(1\: - \:{\rm{PVE}})\: \times \:\:{\rm{k}}}}$$

(n: the adequate sample size in the exposure GWAS; k: the number of variants included in the IV model.)

### Statistical analysis

To pool IV ratio estimates from all the exposure-related SNPs, we utilized the Inverse Variance Weighted (IVW) meta-analysis using a random-effects model. Moreover, Weighted Median, MR-Egger Regression, and weighted modes were employed to verify the IVW findings. The IVW method served as the primary analytical approach, while MR-Egger regression and Weighted Median method were used as supplementary methods to support the IVW estimation. IVW calculated the Wald ratio for each IV between exposure and outcome and performing a meta-analysis of all Wald ratios. Weighted Median method provides a reasonably accurate causal effect estimate even with 50% invalid IVs, though with increased error. MR Egger detects and corrects bias from horizontal pleiotropy, using the regression slope of causal effect and IV strength as the estimate, but it is less statistically powerful.

We also used false discovery rate (FDR) correction because multiplex testing increases the likelihood of type I errors [26]. we performed a Steiger test for each MR analysis, where a *P* < 0.05 supports the hypothesized direction. To examine the possibility of heterogeneity and directional pleiotropy, we used the Cochrane’s *Q* test and the MR-Egger intercept, respectively. A leave-one-out sensitivity analysis was likewise conducted. The MR-PRESSO analysis was used to identify anomalies and address horizontal pleiotropy. All analyses were carried out using R version 4.3.1 with the “TwoSampleMR” and “MRPRESSO” packages.

## Results

### Characteristics of selected genetic variants

According to the predetermined criteria, selected SNPs associated with frailty and neuroticism were presented in Supplementary Tables 1 and 2. The F-statistics were all greater than 10, indicating a low risk of bias due to weak instruments in MR analyses.

### The causal effect of neuroticism on frailty

**Fig. 2:**
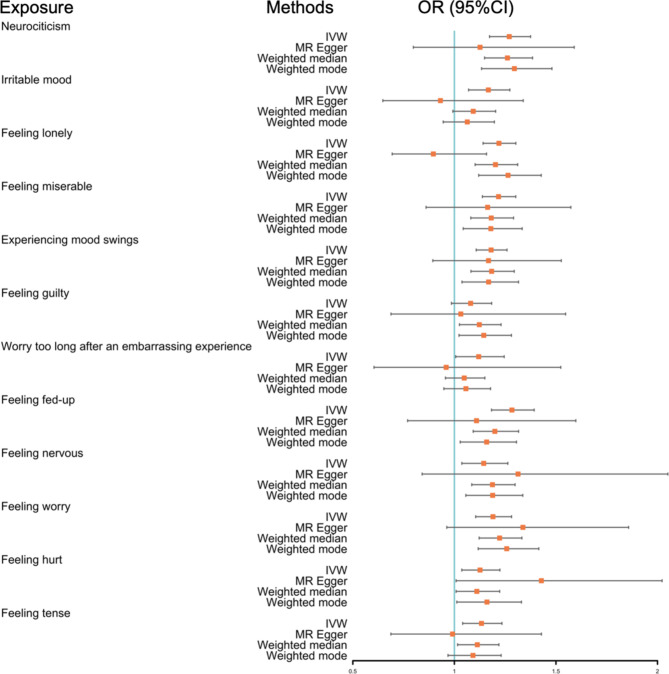
Forest plot of the associations between neuroticism-related phenotypes and frailty of IVW method, MR Egger, weighted median, and weighted mode. Each row represents a trait of neuroticism, with associated odds ratios (ORs) with 95% confidence intervals (CIs)

Using MR analysis, we observed causal effects of neuroticism on the risk of frailty. The results of MR analysis of four methods and different neuroticism-related traits are presented in Table 1; Fig. 2. Genetically predicted neuroticism was associated with a higher FI (OR = 1.627, 95% CI = 1.538–1.722, *P* < 0.001). Ten neuroticism phenotypes showed a positive effect on frailty with the inverse variance weighted (IVW) method, except “worry too long after an embarrassing experience”. Among neuroticism phenotypes, “experiencing mood swings” was assumed as the highest risk factor (OR = 1.756, 95% CI = 1.618–1.905, *P* < 0.001). According to the global test of MR-PRESSO, outlier SNPs were found in 3 (“Feeling lonely”, “Worry too long after an embarrassing experience”, and “Feeling tense”) of the 12 traits. This association remained consistent after correcting for outliers.

**Table 1:** Mendelian randomization results for the associations between frailty-related phenotypes and frailty index of IVW method and MR-PRESSO

Exposure	Outcome	β	IVW method			MR-PRESSO	
			OR (95% CI)	*P*	*P*_FDR_	OR (95% CI)	*P*
Neuroticism	Frailty	0.487	1.627 (1.538–1.722)	< 0.001	< 0.001		
Irritable mood		0.292	1.339 (1.216–1.474)	< 0.001	< 0.001		
Feeling lonely		0.371	1.450 (1.029–2.042)	0.034	0.037	1.640 (1.284–2.095)	0.017
Feeling miserable		0.462	1.588 (1.447–1.743)	< 0.001	< 0.001		
Experiencing mood swings		0.563	1.756 (1.618–1.905)	< 0.001	< 0.001		
Feeling guilty		0.495	1.640 (1.389–1.941)	< 0.001	< 0.001		
Worry too long after an embarrassing experience		0.181	1.198 (0.973, 1.475)	0.088	0.088	1.276 (1.067–1.525)	0.017
Feeling fed-up		0.442	1.555 (1.391–1.740)	< 0.001	< 0.001		
Feeling nervous		0.257	1.293 (1.143–1.463)	< 0.001	< 0.001		
Feeling worry		0.363	1.437 (1.321–1.563)	< 0.001	< 0.001		
Feeling hurt		0.405	1.500 (1.338–1.681)	< 0.001	< 0.001		
Feeling tense		0.304	1.355 (1.176–1.561)	< 0.001	< 0.001	1.408 (1.244–1.592)	< 0.001

IVW: inverse-variance-weighted; OR: odds ratio; CI: Confidence interval; FDR: false discovery rate

Supplementary Fig. 1 shows the forest plots of the individual SNP and combined effects of neuroticism on frailty. The results were congruence between the MR-Egger, weighted, and weighted median mode estimations (Supplementary Table 1). All included SNPs are detailed in Supplementary Table 2. The scatter plots of potential SNP effects on neuroticism versus frailty are shown in Supplementary Fig. 2, where the slope of each plot is the effect size estimate per method. The funnel plots and leave-one-out analysis plots for each exposure-outcome pair are shown in Supplementary Figs. 3 and 4.

The MR-Egger intercept showed no horizontal pleiotropy in all analyses, although heterogeneity was detected for some associations by Cochran’s *Q* statistic (Table 2). Steiger tests suggested that the causal relationships identified were not biased by reverse causation.

**Table 2:** Heterogeneity and pleiotropy analyses of neuroticism on frailty index

Exposure	Outcome	Cochran’s Q test		Egger test	
		Q value	*P*	Egger intercept	*P*
Neuroticism	Frailty	115.545	0.013	0.001	0.713
Irritable mood		45.884	0.053	-0.003	0.665
Feeling lonely		16.244	0.006	-0.011	0.594
Feeling miserable		37.201	0.171	-0.001	0.789
Experiencing mood swings		36.728	0.300	0.003	0.479
Feeling guilty		22.363	0.034	0.010	0.301
Worry too long after an embarrassing experience		57.574	0.000	0.005	0.586
Feeling fed-up		32.302	0.072	0.005	0.318
Feeling nervous		75.294	0.000	-0.004	0.513
Feeling worry		40.428	0.208	-0.002	0.638
Feeling hurt		38.780	0.029	0.000	0.937
Feeling tense		31.461	0.025	-0.004	0.645

### The causal effect of frailty on neuroticism

**Fig. 3:**
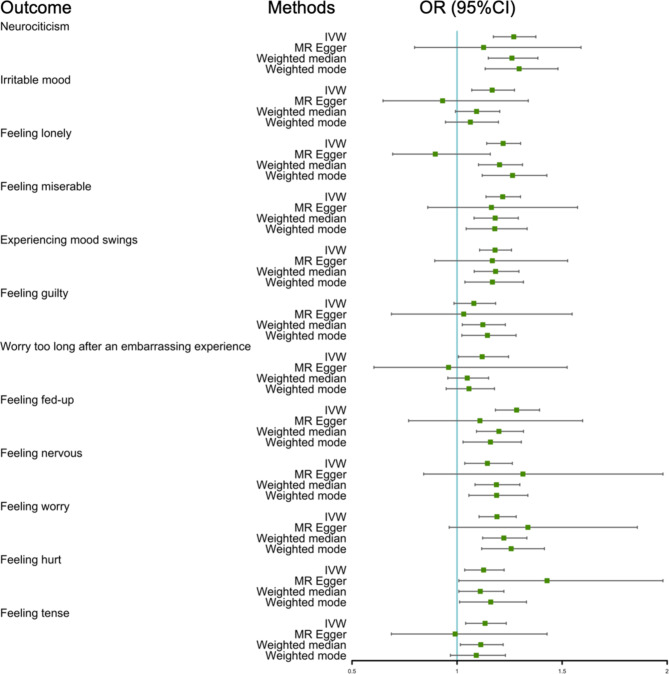
Forest plot of the associations between frailty and neuroticism-related phenotypes of IVW method, MR Egger, weighted median, and weighted mode. Each row represents a trait of neuroticism, with associated odds ratios (ORs) with 95% confidence intervals (CIs)

In the reverse direction, the random-effect IVW methods provided evidence of the causal effect of frailty on a higher risk of neuroticism (OR = 1.270, 95% CI = 1.173–1.375, *P* < 0.001); this causal association was also supported by the weighted, weighted median, and MR-PRESSO methods (Table 3; Fig. 3, Supplementary Table 3). Genetic liability to frailty showed a significant association with a higher risk of most of the neuroticism-related traits except “feeling guilty”. The highest OR of eleven neuroticism-related traits regarding frailty’s impact on “feeling fed-up” risk was estimated at 1.283 (95% CI = 1.183–1.393, *P* < 0.001). Besides, the global test of MR-PRESSO showed outlier SNPs were found in 3 (“Feeling guilty”, “Worry too long after an embarrassing experience”, “Feeling nervous”) of the 12 traits in the MR analysis. After excluding the outlier, the MR-PRESSO method demonstrated a positive effect of frailty on “feeling guilty” (OR = 1.116, 95% CI = 1.042–1.196, *P* = 0.011).

**Table 3:** Mendelian randomization results for the associations between frailty index and frailty-related phenotypes of IVW method and MR-PRESSO

Exposure	Outcome	β	IVW method			MR-PRESSO	
			OR (95% CI)	*P*	*P*_FDR_	OR (95% CI)	*P*
Frailty	Neuroticism	0.239	1.270 (1.173–1.375)	< 0.001	< 0.001		
	Irritable mood	0.155	1.167 (1.070–1.259)	0.001	0.001		
	Feeling lonely	0.198	1.219 (1.141–1.303)	< 0.001	< 0.001		
	Feeling miserable	0.197	1.217 (1.138–1.302)	< 0.001	< 0.001		
	Experiencing mood swings	0.166	1.181 (1.107–1.259)	< 0.001	< 0.001		
	Feeling guilty	0.077	1.080 (0.986–1.183)	0.099	0.099	1.116 (1.042–1.196)	0.011
	Worry too long after an embarrassing experience	0.113	1.120 (1.007–1.259)	0.037	0.041	1.151 (1.047–1.265)	0.014
	Feeling fed-up	0.249	1.283 (1.183–1.393)	< 0.001	< 0.001		
	Feeling nervous	0.135	1.144 (1.037–1.263)	0.007	0.009	1.152 (1.066–1.245)	0.004
	Feeling worry	0.174	1.190 (1.105–1.282)	< 0.001	< 0.001		
	Feeling hurt	0.119	1.126 (1.037–1.224)	0.005	0.007		
	Feeling tense	0.125	1.133 (1.041–1.234)	0.004	0.006		

IVW: inverse-variance-weighted; OR: odds ratio; CI: Confidence interval; FDR: false discovery rate

The forest plots illustrating the individual SNP effects and the combined effects of frailty on neuroticism are shown in Supplementary Fig. 5. SNPs included in the analysis are provided in Supplementary Table 4. The scatter plots, funnel plots, and leave-one-out analysis plots for each exposure-outcome pair are presented in Supplementary Figs. 6–8.

MR Egger intercept showed a horizontal pleiotropy in frailty - “feeling lonely” pair, which was not observed in Cochran’s *Q* test. In other pairs, MR Egger intercept showed no horizontal pleiotropy, although Cochran’s *Q* showed heterogeneity for some associations (Table 4). According to the Steiger tests, the causal relationships identified were not biased by reverse causation.

**Table 4:** Heterogeneity and pleiotropy analyses of neuroticism on frailty index

Exposure	Outcome	Cochran’s Q test		Egger test	
		Q value	*P*	Egger intercept	*P*
Neuroticism	Frailty	18.351	0.074	0.003	0.498
Irritable mood		24.405	0.018	0.005	0.236
Feeling lonely		16.820	0.208	0.007	0.033
Feeling miserable		13.104	0.287	0.001	0.770
Experiencing mood swings		8.091	0.620	0.000	0.936
Feeling guilty		23.856	0.013	0.001	0.825
Worry too long after an embarrassing experience		36.421	0.000	0.004	0.515
Feeling fed-up		25.603	0.019	0.003	0.436
Feeling nervous		37.151	0.000	-0.003	0.545
Feeling worry		18.271	0.108	-0.003	0.489
Feeling hurt		22.861	0.029	-0.005	0.197
Feeling tense		20.401	0.040	0.003	0.476

## Discussion

In this bidirectional MR study, we first discovered a two-way causal relationship between neuroticism and frailty. On the one hand, we identified the positive causal effect of neuroticism on frailty. On the other hand, our reverse analyses revealed that frailty was also positively correlated with neuroticism.

A few epidemiology studies have explored the relationship between neuroticism and frailty. Neuroticism is associated with a wide range of physical conditions, such as cardiovascular disease, impaired immune function, asthma, Parkinson’s disease, and even an increased risk of death [27–31]. It was a strong negative predictor of health, particularly when considering mental health and health behaviors [32]. A longitudinal study involving 5,314 individuals aged 60 and over found that higher levels of neuroticism may be a risk factor for the onset or progression of frailty [14]. Hilda et al. found neuroticism in midlife predicts frailty in later life, with both environmental and genetic factors contributing to this association [15]. High neuroticism is also linked to poor diet, lack of exercise, poor physical health, and procrastination [33]. Hence, the relationship between neuroticism and physical problems is also indirect, as neuroticism provides a vulnerability for developing disease [34]. While neuroticism may have a small positive affect on health behavior through vigilance, the effect is limited [35]. As for the two negative results of the IVW method, worrying too long after an embarrassing experience is a temporary emotional state that is unlikely to affect mental health in a way that contributes to frailty. And guilt is more of a moral feeling than other phenotypes, so frailty has less impact on it.

Our study found that frailty can, in turn, affect neuroticism, despite neuroticism being a relatively stable and genetically determined personality trait [36]. However, no population-based studies have specifically examined causal relationship between frailty and neuroticism. Similarly, Braude et al. found that living with frailty was associated with psychiatric morbidity and reduced well-being following hospital admission for COVID-19 [37]. Older individuals with pre-frailty syndromes showed an improvement in mood after engaging in physical strength-enhancing exercises [38].

The underlying mechanism linking neuroticism and frailty remains unclear. High neuroticism is significantly associated with increased somatic comorbidity, poorer self-rated health, and reduced energy and physical activity. Ellen et al. recently found a positive association between falls and high neuroticism, which is a common and devastating issue among old adults [39]. Some studies suggest that personality may have a more direct impact on mental health than health behaviors or physical health [32, 40]. Frailty may result in reduced physical and social activity. Additionally, frailty is a predictor of mental disorders, disability, hospitalization, institutionalization, and mortality [1, 41].

Frailty and neuroticism share several physiological factors, including abnormalities in C-reactive protein (CRP), interleukin, granulocyte-macrophage colony-stimulating factor, and tumor necrosis factor [42, 43]. In the MR analysis of neuroticism to frailty, SLC44A5 is the gene nearest to the principal SNP locus, rs17096778 (*P* = 7.60 × 10^− 9^). Studies on the expression of mucosal genes in the digestive system have shown that SLC44A5 is involved in the transport of glucose, other sugars, bile salts, organic acids, metal ions, and amines [44]. Furthermore, in liver tissue, SLC44A5 can inhibit cells at G1 phase of the cell cycle by reducing the expression of cell cycle markers, including proliferating cell nuclear antigen and cyclin-dependent kinase 2 [45]. In the analysis of frailty on neuroticism, one of the leading SNPs is rs2071207 (*P* = 1.47 × 10^− 8^), with the nearest gene being RBM5. RBM5 has been found to inhibit cell growth by regulating apoptosis and has shown significant associations with body mass index (BMI), HDL cholesterol, basal metabolic rate, and CRP [46]. Therefore, the bi-directional relationship between neuroticism and frailty is not coincidental, and all these findings support this hypothesis.

Notably, both frailty and neuroticism are associated with a range of adverse outcomes, including reduced quality of life and increased healthcare utilization [1, 2, 47]. Therefore, it is essential to offer timely psychological support and counseling to frail and neurotic patients, or to conduct physical screening for neurotic individuals to prevent a vicious cycle.

As far as we know, this is the first MR study to investigate the causal relationship between neuroticism related traits and frailty. There are several strengths of the study. First, a large sample size and an optimal study design with robust MR and sensitivity methods contribute to the stability of our findings. Second, two distinct approaches were employed to identify potential outliers, which makes the results more reliable. Third, strict criteria for the IVs and the use of bidirectional two-sample MR analyses ensure less confounding bias and exclude the effects of reverse causality. Finally, our analysis stratified neuroticism into eleven specific phenotypes.

Despite these advantages, our study has several limitations. The population analyzed was of European descent, which means the results should be interpreted with caution when applied to other populations. Neuroticism scores and frailty indices were primarily derived from self-report questionnaires, which may not fully reflect objective status of these conditions. Additionally, due to the use of summary data, we were unable to conduct subgroup analysis on urban/rural or gender differences. Furthermore, while sensitivity analyses such as MR-Egger and MR-PRESSO were employed, confounding bias may still persist.

In conclusion, this study supports a bidirectional causal relationship between neuroticism and frailty. Based on our findings, routine frailty screening should be considered for individuals with high neuroticism, and appropriate management of neuroticism is crucial for reducing the risk of frailty. These findings also provide theoretical support for further research on the management of neuroticism to prevent the onset and progression of frailty, and conversely, to explore how managing frailty may mitigate the impact of neuroticism.

## Conclusions

The study showed a positive bi-directional causal relationship between neuroticism-related phenotypes and the risk of frailty from a genetic perspective. Hopefully, our study can illustrate the association between frailty and neuroticism and provide a new sight of possible preventions and interventions for both diseases.

## Electronic supplementary material

Below is the link to the electronic supplementary material.

Supplementary Material 1

Supplementary Material 2

## Data Availability

The data was available in the IEU Open GWAS Project (https://gwas.mrcieu.ac.uk/). These data were derived from the following resources available in the public domain: Frailty index: https://gwas.mrcieu.ac.uk/datasets/ebi-a-GCST90020053/; Neuroticism: https://gwas.mrcieu.ac.uk/datasets/ebi-a-GCST006940/; Irritable mood: https://gwas.mrcieu.ac.uk/datasets/ebi-a-GCST006941/; Feeling lonely: https://gwas.mrcieu.ac.uk/datasets/ebi-a-GCST006942/; Feeling miserable: https://gwas.mrcieu.ac.uk/datasets/ebi-a-GCST006943/; Experiencing mood swings: https://gwas.mrcieu.ac.uk/datasets/ebi-a-GCST006944/; Feeling guilty : https://gwas.mrcieu.ac.uk/datasets/ebi-a-GCST006945/; Worry too long after an embarrassing experience : https://gwas.mrcieu.ac.uk/datasets/ebi-a-GCST006946/; Feeling fed-up : https://gwas.mrcieu.ac.uk/datasets/ebi-a-GCST006947/; Feeling nervous : https://gwas.mrcieu.ac.uk/datasets/ebi-a-GCST006948/; Feeling worry: https://gwas.mrcieu.ac.uk/datasets/ebi-a-GCST006950/; Feeling hurt : https://gwas.mrcieu.ac.uk/datasets/ebi-a-GCST006951/; Feeling tense: https://gwas.mrcieu.ac.uk/datasets/ebi-a-GCST006952/.
