# Supplementary material for: Exploring the bidirectional causality between neuroticism and frailty: a Mendelian randomization analysis
Source: Hereditas. 2025 Jan 25;162:8. doi: 10.1186/s41065-025-00370-2 (PMC11763127; doi:10.1186/s41065-025-00370-2)
Supplement: Supplementary file 1 — Supplementary Material 1 [file 41065_2025_370_MOESM1_ESM.pdf]

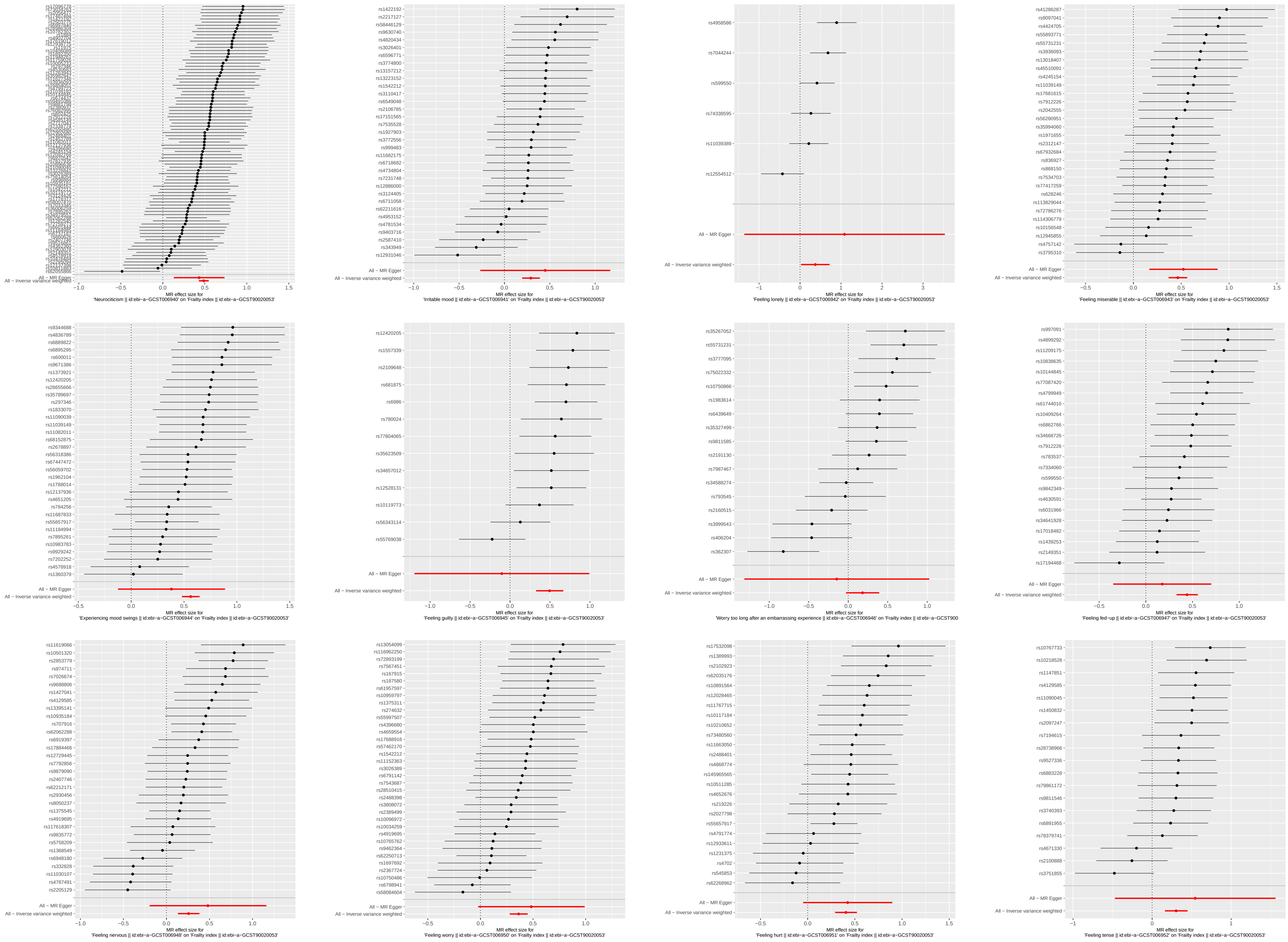

Figure. S1. Forest plot of the individual and combined effect of neuroticism-related phenotype on frailty.

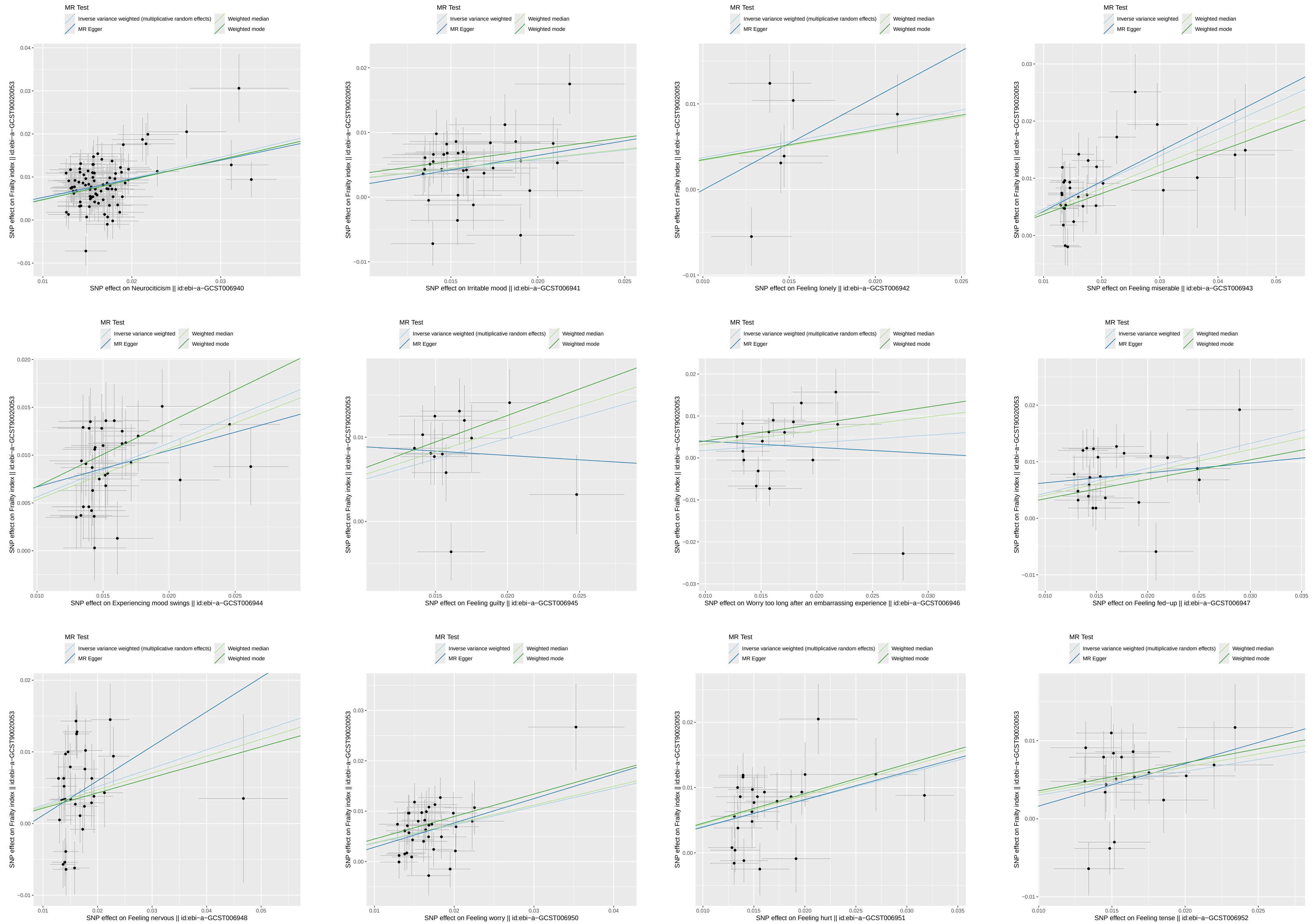

Figure. S2. Scatter plots of SNP effects of neuroticism-related phenotype on frailty, with the slope of each line corresponding to the estimated MR effect per method.

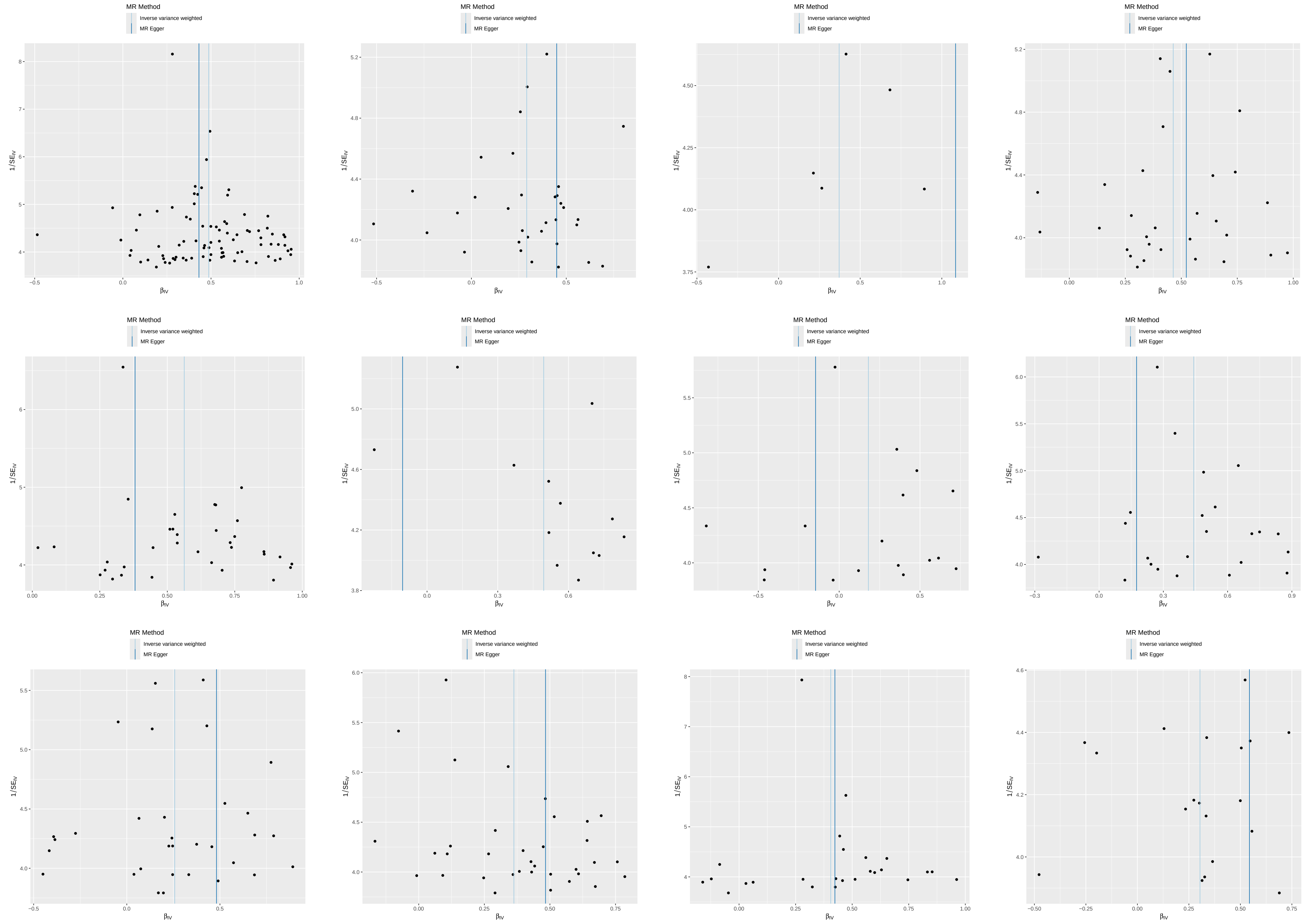

Figure. S3. Funnel plot of neuroticism-related phenotype on frailty.

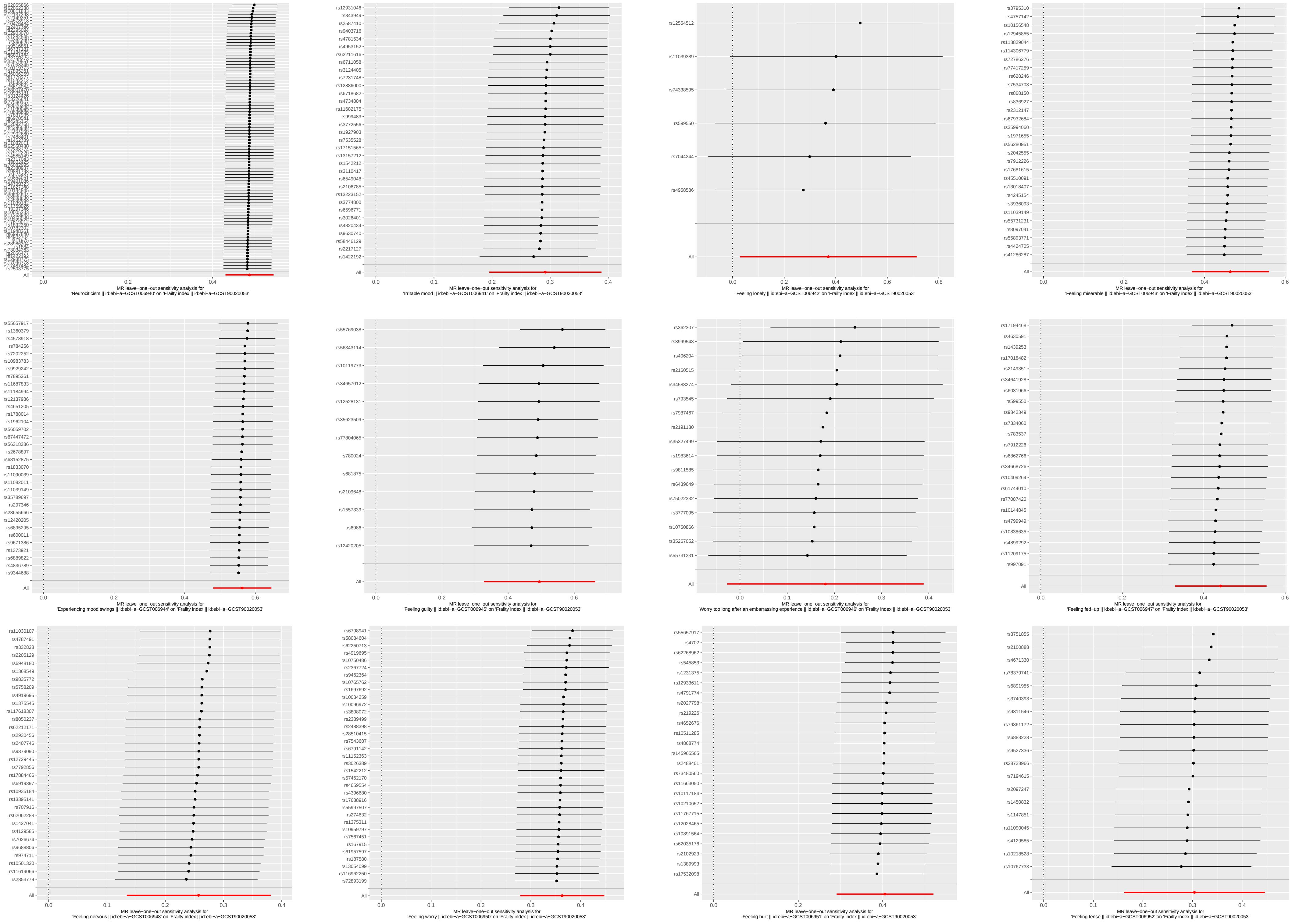

Figure. S4. The leave-one-out estimate of neuroticism-related phenotype on frailty.

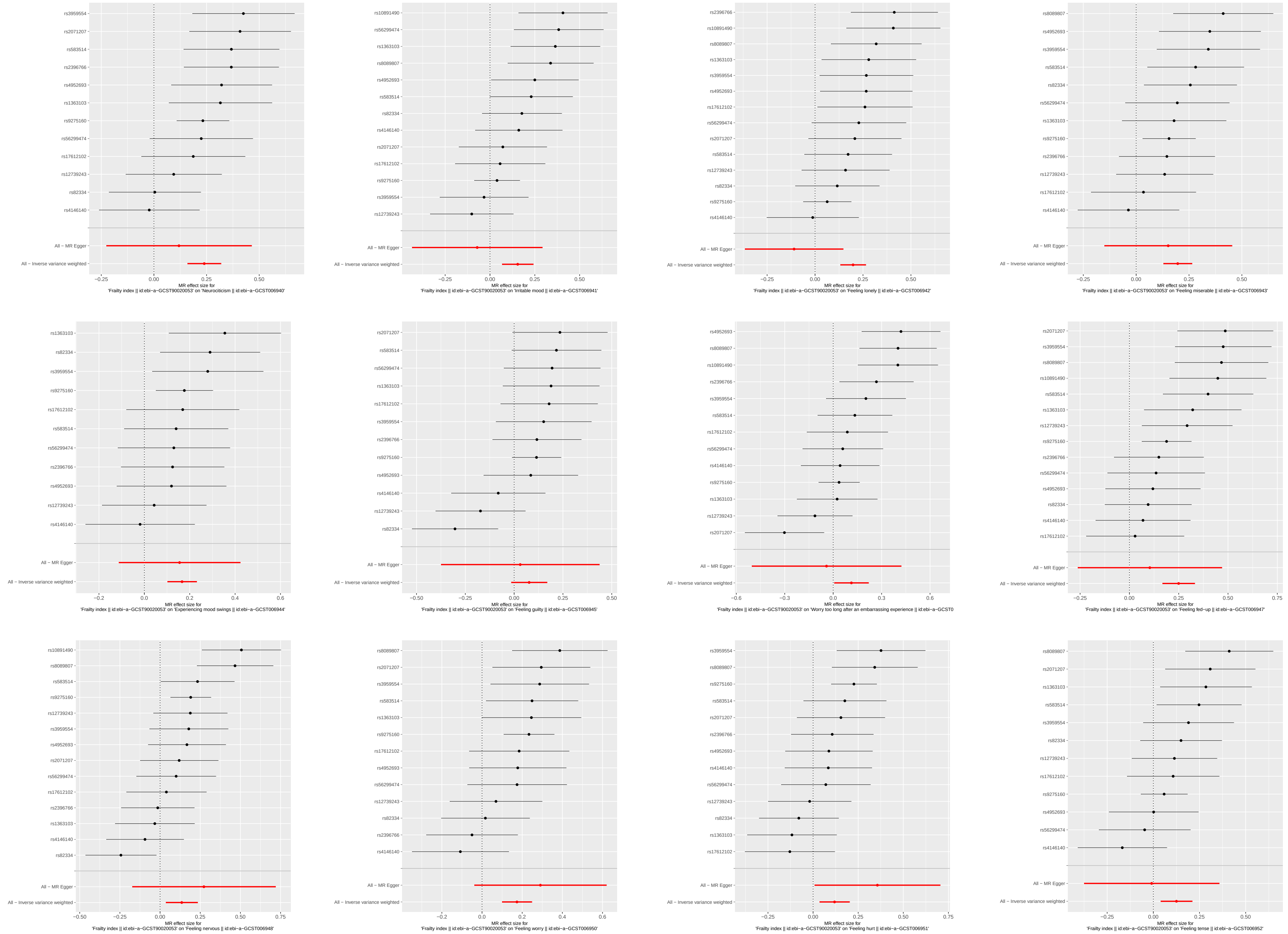

Figure. S5. Forest plot of the individual and combined effect of frailty on neuroticism-related phenotype.

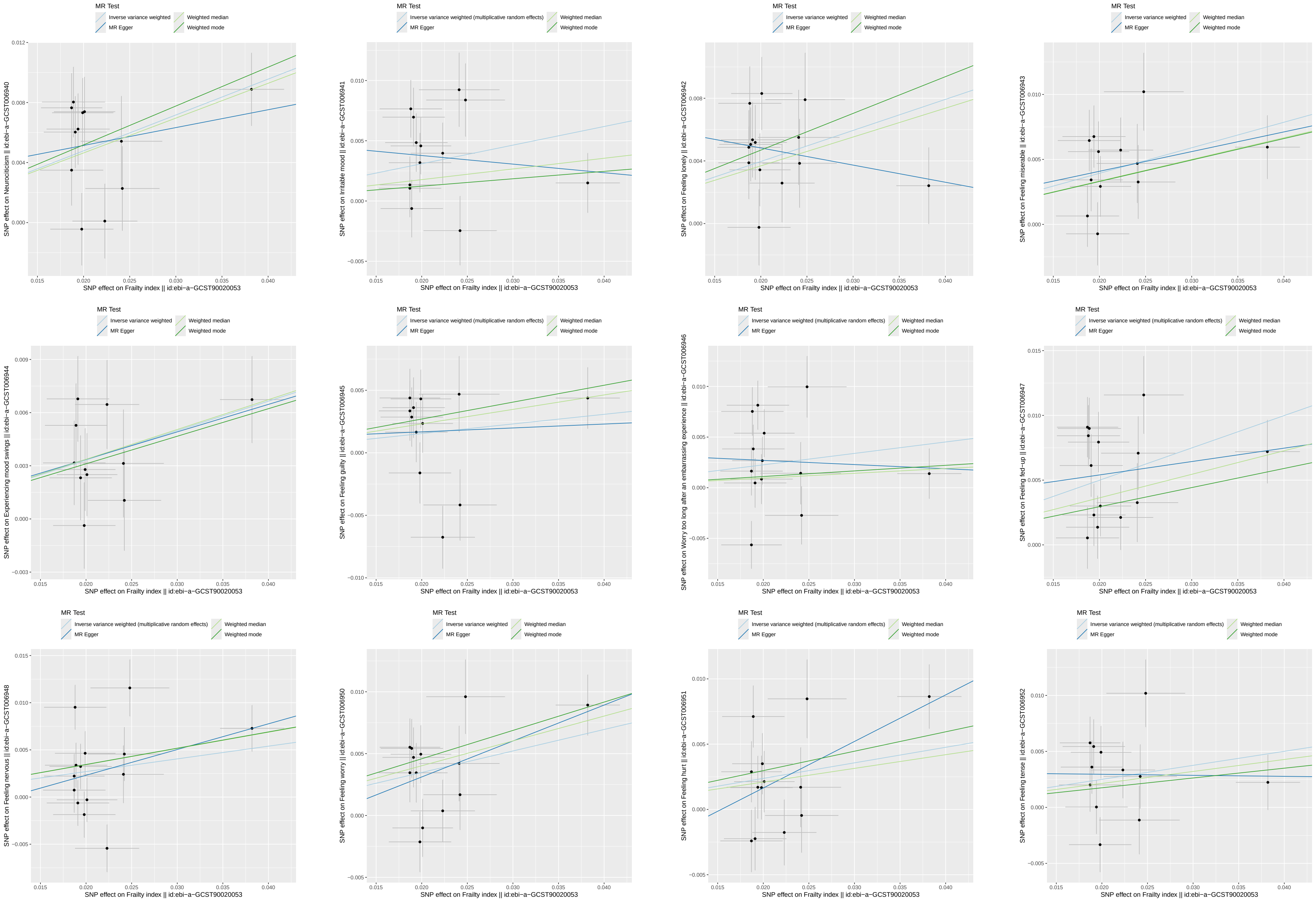

Figure. S6. Scatter plots of SNP effects of frailty on neuroticism-related phenotype, with the slope of each line corresponding to the estimated MR effect per method.

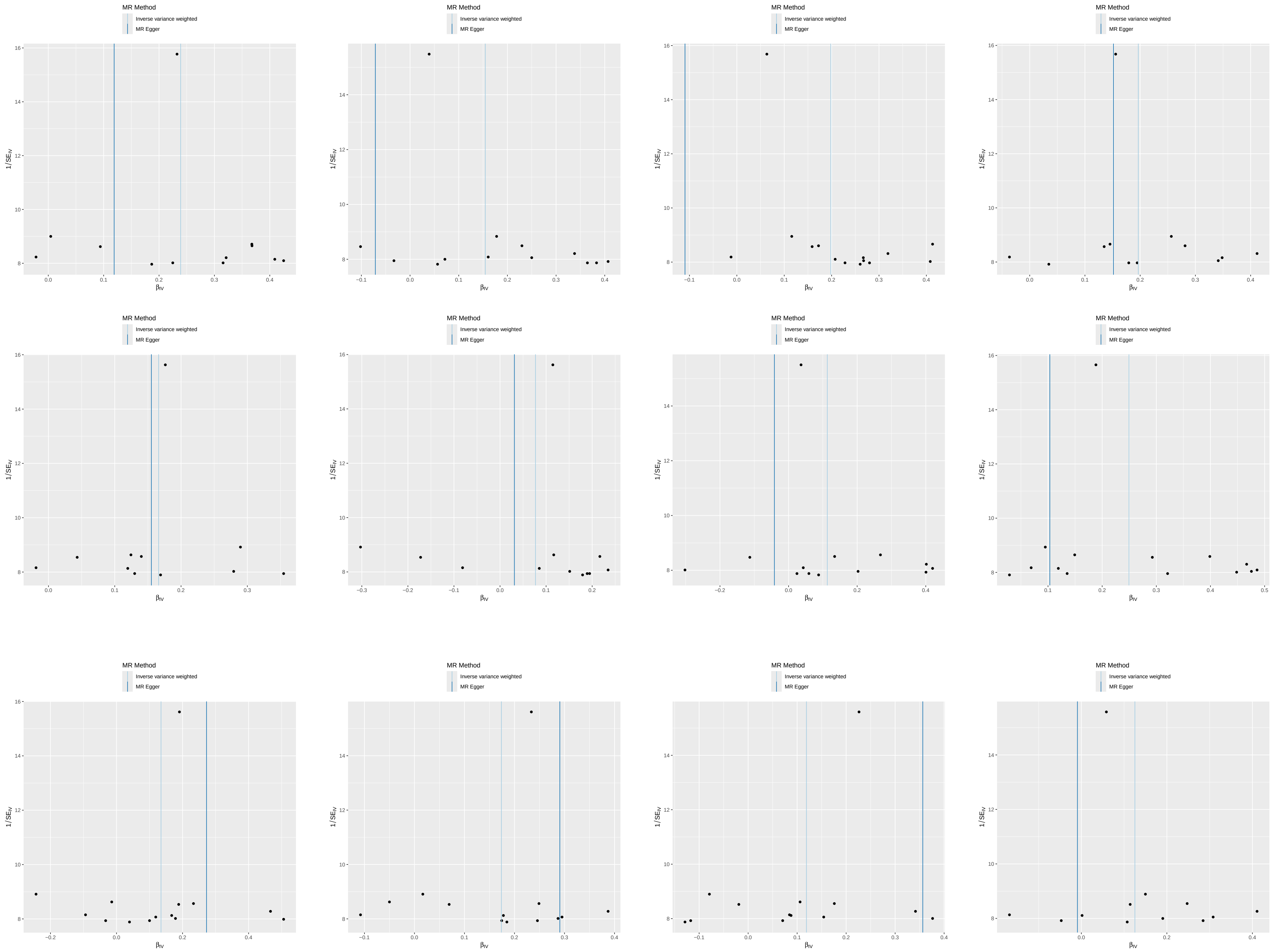

Figure. S7. Funnel plot of frailty on neuroticism-related phenotype.

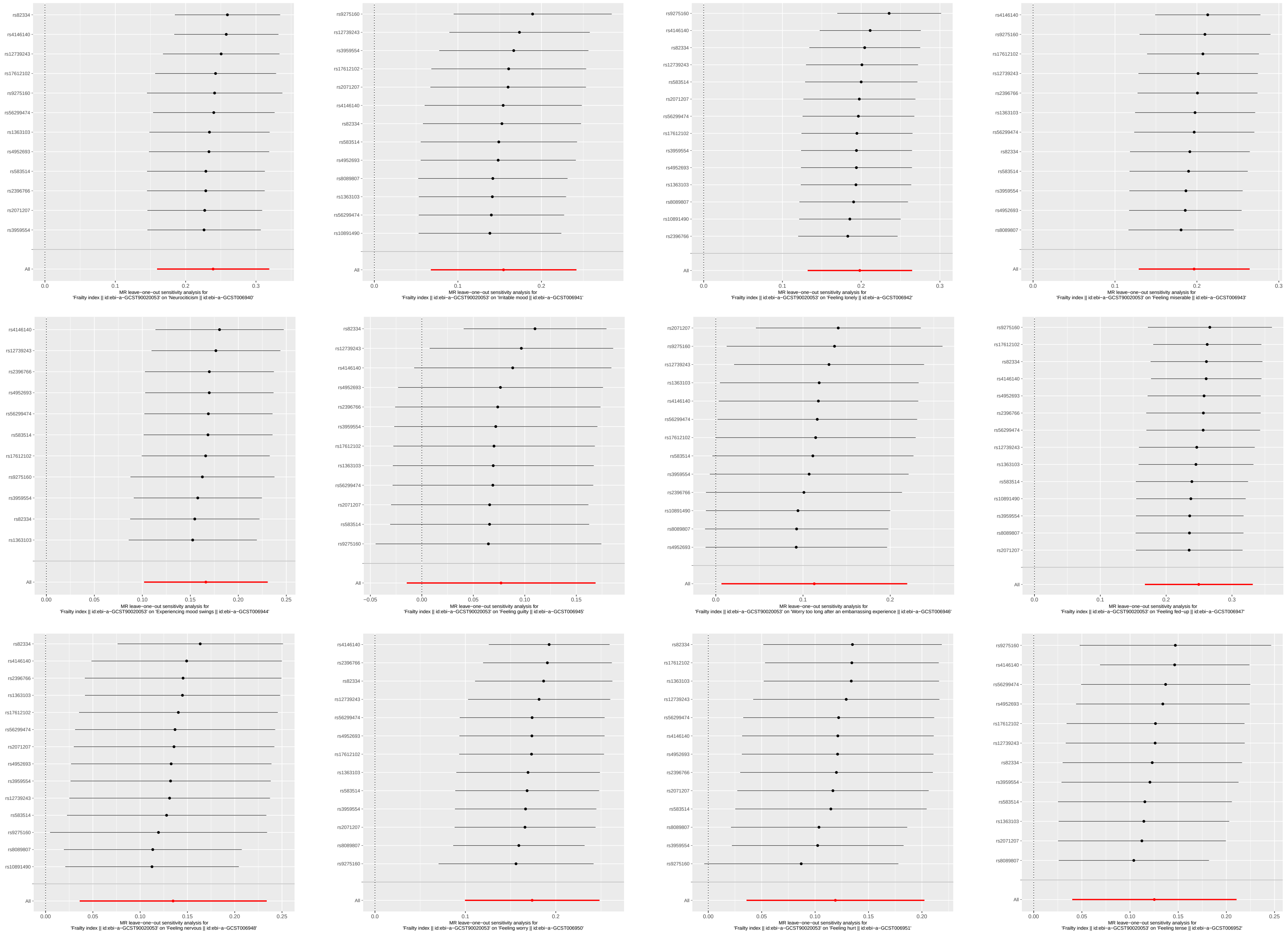

Figure. S8. The leave-one-out estimate of frailty on neuroticism-related phenotype.
