# Supplementary material for: Exploring the bidirectional causality between neuroticism and frailty: a Mendelian randomization analysis
Source: Hereditas. 2025 Jan 25;162:8. doi: 10.1186/s41065-025-00370-2 (PMC11763127; doi:10.1186/s41065-025-00370-2)
Supplement: Supplementary file 2 — Supplementary Material 2 [file 41065_2025_370_MOESM2_ESM.docx]

**Table S1** The effects of neuroticism-related phenotype on frailty.

| **Exposure** | **Outcome** | **Method** | **N of SNP** | **β (95%CI)** | **se** | ***P*** | **OR (95%CI)** | ***P* _fdr_** |
| --- | --- | --- | --- | --- | --- | --- | --- | --- |
| Neuroticism | Frailty | IVW | 85 | 0.487(0.43-0.543) | 0.029 | < 0.001 | 1.627(1.538-1.722) | < 0.001 |
| Irritable mood | Frailty | IVW | 33 | 0.292(0.195-0.388) | 0.049 | < 0.001 | 1.339(1.216-1.474) | < 0.001 |
| Feeling lonely | Frailty | IVW | 6 | 0.371(0.029-0.714) | 0.175 | 0.034 | 1.45(1.029-2.042) | 0.037 |
| Feeling miserable | Frailty | IVW | 31 | 0.462(0.37-0.555) | 0.047 | < 0.001 | 1.588(1.447-1.743) | < 0.001 |
| Experiencing mood swings | Frailty | IVW | 34 | 0.563(0.481-0.645) | 0.042 | < 0.001 | 1.756(1.618-1.905) | < 0.001 |
| Feeling guilty | Frailty | IVW | 13 | 0.495(0.327-0.663) | 0.086 | < 0.001 | 1.64(1.386-1.941) | < 0.001 |
| Worry too long after an embarrassing experience | Frailty | IVW | 17 | 0.181(-0.027-0.389) | 0.106 | 0.088 | 1.198(0.973-1.475) | 0.088 |
| Feeling fed-up | Frailty | IVW | 23 | 0.442(0.33-0.554) | 0.057 | < 0.001 | 1.555(1.391-1.74) | < 0.001 |
| Feeling nervous | Frailty | IVW | 32 | 0.257(0.134-0.38) | 0.063 | < 0.001 | 1.293(1.143-1.463) | < 0.001 |
| Feeling worry | Frailty | IVW | 35 | 0.363(0.279-0.447) | 0.043 | < 0.001 | 1.437(1.321-1.563) | < 0.001 |
| Feeling hurt | Frailty | IVW | 25 | 0.405(0.291-0.519) | 0.058 | < 0.001 | 1.5(1.338-1.681) | < 0.001 |
| Feeling tense | Frailty | IVW | 19 | 0.304(0.162-0.445) | 0.072 | < 0.001 | 1.355(1.176-1.561) | < 0.001 |
| Neuroticism | Frailty | MR Egger | 85 | 0.432(0.132-0.731) | 0.153 | 0.006 | 1.54(1.141-2.078) | 0.036 |
| Irritable mood | Frailty | MR Egger | 33 | 0.45(-0.265-1.165) | 0.365 | 0.227 | 1.568(0.767-3.204) | 0.389 |
| Feeling lonely | Frailty | MR Egger | 6 | 1.084(-1.357-3.526) | 1.246 | 0.433 | 2.957(0.257-33.987) | 0.578 |
| Feeling miserable | Frailty | MR Egger | 31 | 0.506(0.175-0.838) | 0.169 | 0.006 | 1.659(1.191-2.312) | 0.036 |
| Experiencing mood swings | Frailty | MR Egger | 34 | 0.381(-0.124-0.886) | 0.258 | 0.149 | 1.463(0.883-2.424) | 0.346 |
| Feeling guilty | Frailty | MR Egger | 13 | -0.104(-1.197-0.989) | 0.558 | 0.856 | 0.902(0.302-2.689) | 0.856 |
| Worry too long after an embarrassing experience | Frailty | MR Egger | 17 | -0.146(-1.314-1.023) | 0.596 | 0.810 | 0.864(0.269-2.78) | 0.856 |
| Feeling fed-up | Frailty | MR Egger | 23 | 0.175(-0.349-0.699) | 0.267 | 0.519 | 1.191(0.706-2.011) | 0.623 |
| Feeling nervous | Frailty | MR Egger | 32 | 0.481(-0.195-1.158) | 0.345 | 0.173 | 1.618(0.823-3.182) | 0.346 |
| Feeling worry | Frailty | MR Egger | 35 | 0.483(-0.022-0.988) | 0.258 | 0.070 | 1.621(0.978-2.687) | 0.273 |
| Feeling hurt | Frailty | MR Egger | 25 | 0.424(-0.047-0.895) | 0.240 | 0.091 | 1.528(0.954-2.446) | 0.273 |
| Feeling tense | Frailty | MR Egger | 19 | 0.544(-0.471-1.558) | 0.518 | 0.308 | 1.723(0.625-4.751) | 0.462 |
| Neuroticism | Frailty | Weighted median | 85 | 0.468(0.392-0.543) | 0.039 | < 0.001 | 1.597(1.48-1.722) | < 0.001 |
| Irritable mood | Frailty | Weighted median | 33 | 0.297(0.177-0.418) | 0.061 | < 0.001 | 1.346(1.193-1.518) | < 0.001 |
| Feeling lonely | Frailty | Weighted median | 6 | 0.34(0.067-0.613) | 0.139 | 0.015 | 1.405(1.07-1.846) | 0.015 |
| Feeling miserable | Frailty | Weighted median | 31 | 0.408(0.283-0.533) | 0.064 | < 0.001 | 1.504(1.328-1.704) | < 0.001 |
| Experiencing mood swings | Frailty | Weighted median | 34 | 0.534(0.413-0.655) | 0.062 | < 0.001 | 1.705(1.511-1.925) | < 0.001 |
| Feeling guilty | Frailty | Weighted median | 13 | 0.552(0.376-0.727) | 0.090 | < 0.001 | 1.736(1.457-2.069) | < 0.001 |
| Worry too long after an embarrassing experience | Frailty | Weighted median | 17 | 0.327(0.146-0.507) | 0.092 | < 0.001 | 1.386(1.157-1.661) | < 0.001 |
| Feeling fed-up | Frailty | Weighted median | 23 | 0.405(0.262-0.548) | 0.073 | < 0.001 | 1.5(1.299-1.731) | < 0.001 |
| Feeling nervous | Frailty | Weighted median | 32 | 0.235(0.105-0.365) | 0.066 | < 0.001 | 1.265(1.111-1.44) | < 0.001 |
| Feeling worry | Frailty | Weighted median | 35 | 0.373(0.255-0.492) | 0.060 | < 0.001 | 1.453(1.291-1.635) | < 0.001 |
| Feeling hurt | Frailty | Weighted median | 25 | 0.44(0.296-0.584) | 0.074 | < 0.001 | 1.552(1.344-1.793) | < 0.001 |
| Feeling tense | Frailty | Weighted median | 19 | 0.332(0.179-0.485) | 0.078 | < 0.001 | 1.394(1.196-1.624) | < 0.001 |
| Neuroticism | Frailty | Weighted mode | 85 | 0.469(0.293-0.645) | 0.090 | < 0.001 | 1.599(1.341-1.906) | < 0.001 |
| Irritable mood | Frailty | Weighted mode | 33 | 0.369(0.16-0.578) | 0.107 | 0.002 | 1.446(1.173-1.783) | 0.005 |
| Feeling lonely | Frailty | Weighted mode | 6 | 0.348(-0.037-0.733) | 0.196 | 0.136 | 1.416(0.964-2.081) | 0.136 |
| Feeling miserable | Frailty | Weighted mode | 31 | 0.374(0.129-0.619) | 0.125 | 0.006 | 1.453(1.137-1.858) | 0.010 |
| Experiencing mood swings | Frailty | Weighted mode | 34 | 0.672(0.396-0.948) | 0.141 | < 0.001 | 1.958(1.486-2.581) | < 0.001 |
| Feeling guilty | Frailty | Weighted mode | 13 | 0.629(0.277-0.982) | 0.180 | 0.004 | 1.877(1.319-2.67) | 0.009 |
| Worry too long after an embarrassing experience | Frailty | Weighted mode | 17 | 0.405(0.078-0.733) | 0.167 | 0.027 | 1.5(1.081-2.081) | 0.033 |
| Feeling fed-up | Frailty | Weighted mode | 23 | 0.344(0.083-0.605) | 0.133 | 0.017 | 1.411(1.087-1.832) | 0.025 |
| Feeling nervous | Frailty | Weighted mode | 32 | 0.214(-0.041-0.469) | 0.130 | 0.110 | 1.239(0.96-1.599) | 0.120 |
| Feeling worry | Frailty | Weighted mode | 35 | 0.446(0.159-0.734) | 0.147 | 0.004 | 1.563(1.172-2.083) | 0.009 |
| Feeling hurt | Frailty | Weighted mode | 25 | 0.453(0.221-0.686) | 0.119 | 0.001 | 1.573(1.247-1.985) | 0.003 |
| Feeling tense | Frailty | Weighted mode | 19 | 0.359(0.073-0.644) | 0.146 | 0.024 | 1.431(1.076-1.904) | 0.032 |

**Table S2** Characteristics of genetic variants used to estimate the effect of neuroticism-related phenotype on frailty.

| **SNP** | **Exposure** | **Outcome** | **Allele of exposure** | **Allele of outcome** | **chr** | **pos** | **Steiger *P* val** | **F** |
| --- | --- | --- | --- | --- | --- | --- | --- | --- |
| rs10005233 | Neuroticism | Frailty | T | T | 4 | 90743331 | 0.352 | 40.553 |
| rs10119773 | Neuroticism | Frailty | G | G | 9 | 23736400 | 0.015 | 49.066 |
| rs10144845 | Neuroticism | Frailty | T | T | 14 | 75237770 | 0.11 | 54.77 |
| rs10456089 | Neuroticism | Frailty | A | A | 6 | 11959836 | 0.538 | 36.529 |
| rs10476484 | Neuroticism | Frailty | G | G | 5 | 164438264 | 0.002 | 33.924 |
| rs10782302 | Neuroticism | Frailty | C | C | 6 | 100993125 | 0.727 | 30.293 |
| rs10811883 | Neuroticism | Frailty | T | T | 9 | 23291526 | 0 | 53.01 |
| rs10896636 | Neuroticism | Frailty | G | G | 11 | 57448032 | 0.052 | 42.939 |
| rs10935181 | Neuroticism | Frailty | A | A | 3 | 136098408 | 0.02 | 50.871 |
| rs11039182 | Neuroticism | Frailty | C | C | 11 | 47346723 | 0.104 | 57.751 |
| rs11082011 | Neuroticism | Frailty | T | T | 18 | 35145122 | 0.009 | 89.871 |
| rs11090045 | Neuroticism | Frailty | A | A | 22 | 41753603 | 0.023 | 59.55 |
| rs11184985 | Neuroticism | Frailty | C | C | 1 | 107313610 | 0.018 | 30.556 |
| rs11263943 | Neuroticism | Frailty | G | G | 1 | 37198130 | 0.282 | 46.527 |
| rs11627348 | Neuroticism | Frailty | A | A | 14 | 57295404 | 0.267 | 34.311 |
| rs11759026 | Neuroticism | Frailty | G | G | 6 | 126792095 | 0.464 | 30.603 |
| rs11948261 | Neuroticism | Frailty | A | A | 5 | 107753691 | 0.482 | 40.059 |
| rs11975 | Neuroticism | Frailty | C | C | 9 | 96327691 | 0.513 | 43.85 |
| rs12092768 | Neuroticism | Frailty | A | A | 1 | 174432882 | 0.074 | 36.335 |
| rs12137398 | Neuroticism | Frailty | T | T | 1 | 7790548 | 0.001 | 37.983 |
| rs12137936 | Neuroticism | Frailty | G | G | 1 | 72720357 | 0.122 | 30.746 |
| rs1282545 | Neuroticism | Frailty | C | C | 3 | 107199709 | 0.004 | 52.722 |
| rs12902680 | Neuroticism | Frailty | C | C | 15 | 46316484 | 0.125 | 32.301 |
| rs12903078 | Neuroticism | Frailty | A | A | 15 | 47503469 | 0.005 | 30.468 |
| rs12938775 | Neuroticism | Frailty | A | A | 17 | 2574821 | 0.545 | 46.813 |
| rs13226841 | Neuroticism | Frailty | C | C | 7 | 126389408 | 0.018 | 56.119 |
| rs1422192 | Neuroticism | Frailty | A | A | 5 | 87959023 | 0.75 | 38.15 |
| rs1452789 | Neuroticism | Frailty | A | A | 18 | 53115879 | 0.074 | 42.753 |
| rs1542212 | Neuroticism | Frailty | G | G | 3 | 35683935 | 0.021 | 46.178 |
| rs16854051 | Neuroticism | Frailty | T | T | 4 | 42123892 | 0.274 | 31.179 |
| rs17096778 | Neuroticism | Frailty | G | G | 1 | 75809970 | 0.969 | 33.805 |
| rs17487484 | Neuroticism | Frailty | G | G | 18 | 50723283 | 0.839 | 38.634 |
| rs17619012 | Neuroticism | Frailty | T | T | 2 | 63893589 | 0.638 | 31.544 |
| rs1778377 | Neuroticism | Frailty | T | T | 14 | 41694018 | 0.029 | 36.728 |
| rs1884 | Neuroticism | Frailty | C | C | 11 | 112880597 | 0.629 | 40.163 |
| rs1892350 | Neuroticism | Frailty | G | G | 13 | 68080614 | 0.497 | 38.253 |
| rs2056477 | Neuroticism | Frailty | C | C | 7 | 2079744 | 0.907 | 33.352 |
| rs2149351 | Neuroticism | Frailty | G | G | 9 | 120501644 | 0 | 48.126 |
| rs2295094 | Neuroticism | Frailty | A | A | 20 | 33447915 | 0.002 | 32.733 |
| rs2380937 | Neuroticism | Frailty | C | C | 9 | 4145781 | 0.185 | 32.248 |
| rs2407746 | Neuroticism | Frailty | G | G | 8 | 4937757 | 0.002 | 49.038 |
| rs2488401 | Neuroticism | Frailty | T | T | 1 | 197702401 | 0.099 | 37.366 |
| rs2503775 | Neuroticism | Frailty | G | G | 6 | 98521600 | 0.757 | 41.252 |
| rs2717043 | Neuroticism | Frailty | T | T | 2 | 58179019 | 0.119 | 40.915 |
| rs28986304 | Neuroticism | Frailty | C | C | 6 | 29566082 | 0.677 | 37.796 |
| rs297346 | Neuroticism | Frailty | G | G | 11 | 16355771 | 0.286 | 42.882 |
| rs3026389 | Neuroticism | Frailty | G | G | 11 | 31813529 | 0.044 | 38.196 |
| rs3124426 | Neuroticism | Frailty | T | T | 13 | 55937346 | 0.043 | 31.663 |
| rs34979551 | Neuroticism | Frailty | G | G | 6 | 100848871 | 0.029 | 30.44 |
| rs35982947 | Neuroticism | Frailty | C | C | 17 | 38214275 | 0.348 | 32.878 |
| rs36006259 | Neuroticism | Frailty | T | T | 14 | 30268871 | 0.031 | 32.387 |
| rs3936093 | Neuroticism | Frailty | G | G | 15 | 78101909 | 0.216 | 41.106 |
| rs4245154 | Neuroticism | Frailty | G | G | 11 | 113388674 | 0.017 | 71.747 |
| rs4362360 | Neuroticism | Frailty | C | C | 15 | 86940622 | 0.008 | 30.407 |
| rs4396680 | Neuroticism | Frailty | G | G | 2 | 10178236 | 0.099 | 34.662 |
| rs4530683 | Neuroticism | Frailty | G | G | 4 | 139053569 | 0.377 | 30.838 |
| rs4578918 | Neuroticism | Frailty | C | C | 20 | 44721656 | 0.001 | 41.717 |
| rs4585149 | Neuroticism | Frailty | C | C | 3 | 157493952 | 0.173 | 34.081 |
| rs4799723 | Neuroticism | Frailty | G | G | 18 | 31367050 | 0.23 | 37.491 |
| rs4902704 | Neuroticism | Frailty | C | C | 14 | 69703588 | 0.648 | 35.791 |
| rs58007470 | Neuroticism | Frailty | T | T | 5 | 153666790 | 0.044 | 31.186 |
| rs59491086 | Neuroticism | Frailty | G | G | 2 | 157132879 | 0.138 | 44.084 |
| rs62055866 | Neuroticism | Frailty | C | C | 16 | 30969557 | 0.075 | 39.655 |
| rs62062288 | Neuroticism | Frailty | A | A | 17 | 44096553 | 0 | 142.01 |
| rs62550480 | Neuroticism | Frailty | T | T | 9 | 11449346 | 0.099 | 42.013 |
| rs6601444 | Neuroticism | Frailty | T | T | 8 | 10211175 | 0.016 | 32.191 |
| rs6737187 | Neuroticism | Frailty | A | A | 2 | 226360417 | 0.015 | 31.858 |
| rs674437 | Neuroticism | Frailty | A | A | 11 | 88689953 | 0.165 | 40.028 |
| rs6970541 | Neuroticism | Frailty | G | G | 7 | 38963191 | 0.085 | 34.582 |
| rs6997840 | Neuroticism | Frailty | C | C | 8 | 141658361 | 0.798 | 31.206 |
| rs7033345 | Neuroticism | Frailty | C | C | 9 | 28717573 | 0.024 | 36.013 |
| rs72759273 | Neuroticism | Frailty | G | G | 9 | 126973873 | 0.025 | 29.847 |
| rs73034263 | Neuroticism | Frailty | G | G | 11 | 133808038 | 0.991 | 31.134 |
| rs7338774 | Neuroticism | Frailty | G | G | 13 | 69344134 | 0.144 | 36.846 |
| rs75614054 | Neuroticism | Frailty | T | T | 9 | 98275789 | 0.014 | 58.47 |
| rs76082995 | Neuroticism | Frailty | C | C | 16 | 87426307 | 0.163 | 34.435 |
| rs77580167 | Neuroticism | Frailty | T | T | 5 | 166208982 | 0.066 | 30.515 |
| rs7837935 | Neuroticism | Frailty | G | G | 8 | 65562019 | 0.094 | 31.489 |
| rs7895261 | Neuroticism | Frailty | G | G | 10 | 58751054 | 0.028 | 31.634 |
| rs7912226 | Neuroticism | Frailty | A | A | 10 | 107652144 | 0.18 | 31.963 |
| rs802425 | Neuroticism | Frailty | T | T | 7 | 86294952 | 0.182 | 32.416 |
| rs860626 | Neuroticism | Frailty | G | G | 10 | 119301703 | 0.008 | 36.924 |
| rs9516861 | Neuroticism | Frailty | A | A | 13 | 97854889 | 0.011 | 31.352 |
| rs9881798 | Neuroticism | Frailty | C | C | 3 | 16846967 | 0.117 | 45.81 |
| rs998884 | Neuroticism | Frailty | G | G | 2 | 148539852 | 0.013 | 57.723 |
| rs11682175 | Irritable mood | Frailty | C | C | 2 | 57987593 | 0.019 | 32.792 |
| rs12886000 | Irritable mood | Frailty | T | T | 14 | 98501877 | 0.02 | 30.903 |
| rs12931046 | Irritable mood | Frailty | A | A | 16 | 30987144 | 0.119 | 33.818 |
| rs13157212 | Irritable mood | Frailty | C | C | 5 | 46055494 | 0.097 | 29.892 |
| rs13223152 | Irritable mood | Frailty | G | G | 7 | 69948241 | 0.062 | 37.53 |
| rs1422192 | Irritable mood | Frailty | A | A | 5 | 87959023 | 0.412 | 48.316 |
| rs1542212 | Irritable mood | Frailty | G | G | 3 | 35683935 | 0.086 | 31.959 |
| rs17151565 | Irritable mood | Frailty | G | G | 8 | 10133709 | 0.051 | 34.113 |
| rs1927903 | Irritable mood | Frailty | C | C | 9 | 120514954 | 0.036 | 30.057 |
| rs2106785 | Irritable mood | Frailty | T | T | 17 | 43919105 | 0.015 | 53.765 |
| rs2217127 | Irritable mood | Frailty | G | G | 18 | 35221630 | 0.345 | 30.335 |
| rs2587410 | Irritable mood | Frailty | T | T | 18 | 63536017 | 0.013 | 32.799 |
| rs3026401 | Irritable mood | Frailty | T | T | 11 | 31807524 | 0.079 | 37.358 |
| rs3110417 | Irritable mood | Frailty | G | G | 8 | 89476451 | 0.074 | 34.059 |
| rs3124405 | Irritable mood | Frailty | T | T | 13 | 55970997 | 0.004 | 41.994 |
| rs343949 | Irritable mood | Frailty | T | T | 2 | 44973791 | 0.017 | 37.765 |
| rs3772556 | Irritable mood | Frailty | T | T | 3 | 105249211 | 0.028 | 31.476 |
| rs3774800 | Irritable mood | Frailty | A | A | 3 | 49334768 | 0.072 | 36.705 |
| rs4734804 | Irritable mood | Frailty | G | G | 8 | 105696848 | 0.022 | 31.129 |
| rs4781534 | Irritable mood | Frailty | C | C | 16 | 13805594 | 0.002 | 31.15 |
| rs4820434 | Irritable mood | Frailty | T | T | 22 | 41637119 | 0.153 | 34.433 |
| rs4953152 | Irritable mood | Frailty | A | A | 2 | 45170153 | 0.001 | 37.596 |
| rs58446129 | Irritable mood | Frailty | T | T | 13 | 66582410 | 0.216 | 31.668 |
| rs62211616 | Irritable mood | Frailty | A | A | 20 | 33398508 | 0.001 | 41.182 |
| rs6549048 | Irritable mood | Frailty | A | A | 3 | 85733205 | 0.064 | 36.088 |
| rs6596771 | Irritable mood | Frailty | A | A | 5 | 107512536 | 0.081 | 35.881 |
| rs6711058 | Irritable mood | Frailty | A | A | 2 | 205116051 | 0.007 | 36.623 |
| rs6718682 | Irritable mood | Frailty | T | T | 2 | 122663627 | 0.012 | 36.932 |
| rs7231748 | Irritable mood | Frailty | G | G | 18 | 53109035 | 0.004 | 47.405 |
| rs7535528 | Irritable mood | Frailty | A | A | 1 | 2444414 | 0.05 | 32.263 |
| rs9403716 | Irritable mood | Frailty | A | A | 6 | 100825710 | 0.002 | 34.177 |
| rs9630740 | Irritable mood | Frailty | G | G | 17 | 26242756 | 0.174 | 33.158 |
| rs999483 | Irritable mood | Frailty | G | G | 9 | 135301389 | 0.005 | 49.478 |
| rs11039389 | Feeling lonely | Frailty | C | C | 11 | 47796062 | 0.008 | 35.673 |
| rs12554512 | Feeling lonely | Frailty | C | C | 9 | 23352293 | 0.081 | 29.912 |
| rs4958586 | Feeling lonely | Frailty | A | A | 5 | 152248567 | 0.787 | 34.096 |
| rs599550 | Feeling lonely | Frailty | A | A | 18 | 53252388 | 0.036 | 42.947 |
| rs7044244 | Feeling lonely | Frailty | A | A | 9 | 96397689 | 0.289 | 41.134 |
| rs74338595 | Feeling lonely | Frailty | C | C | 2 | 212749786 | 0.019 | 33.248 |
| rs10156548 | Feeling miserable | Frailty | C | C | 9 | 23318433 | 0.003 | 40.46 |
| rs11039149 | Feeling miserable | Frailty | G | G | 11 | 47276675 | 0.142 | 54.189 |
| rs113829044 | Feeling miserable | Frailty | C | C | 11 | 27664695 | 0.02 | 33.819 |
| rs114306779 | Feeling miserable | Frailty | T | T | 3 | 35240632 | 0.023 | 30.586 |
| rs12094143 | Feeling miserable | Frailty | G | G | 1 | 174481124 | 0.054 | 32.212 |
| rs12945855 | Feeling miserable | Frailty | C | C | 17 | 16139149 | 0.005 | 33.645 |
| rs13018407 | Feeling miserable | Frailty | C | C | 2 | 172973900 | 0.356 | 30.721 |
| rs17681615 | Feeling miserable | Frailty | A | A | 18 | 50124619 | 0.17 | 35.144 |
| rs1971655 | Feeling miserable | Frailty | T | T | 8 | 1519472 | 0.067 | 31.532 |
| rs2042555 | Feeling miserable | Frailty | A | A | 2 | 148555489 | 0.166 | 31.581 |
| rs2312147 | Feeling miserable | Frailty | C | C | 2 | 58222928 | 0.016 | 53.924 |
| rs35994060 | Feeling miserable | Frailty | T | T | 17 | 2589488 | 0.032 | 45.761 |
| rs3795310 | Feeling miserable | Frailty | T | T | 1 | 8431607 | 0.003 | 37.424 |
| rs3936093 | Feeling miserable | Frailty | G | G | 15 | 78101909 | 0.341 | 34.448 |
| rs41286287 | Feeling miserable | Frailty | A | A | 6 | 29579991 | 0.932 | 32.818 |
| rs4245154 | Feeling miserable | Frailty | G | G | 11 | 113388674 | 0.244 | 38.826 |
| rs4424705 | Feeling miserable | Frailty | A | A | 11 | 112863579 | 0.736 | 36.968 |
| rs45510091 | Feeling miserable | Frailty | G | G | 4 | 123186393 | 0.321 | 32.61 |
| rs4757142 | Feeling miserable | Frailty | A | A | 11 | 13325695 | 0.005 | 34.099 |
| rs55731231 | Feeling miserable | Frailty | G | G | 18 | 35183274 | 0.386 | 40.473 |
| rs55893771 | Feeling miserable | Frailty | T | T | 7 | 2096255 | 0.392 | 47.774 |
| rs56280951 | Feeling miserable | Frailty | A | A | 17 | 44041107 | 0.031 | 51.886 |
| rs628246 | Feeling miserable | Frailty | C | C | 11 | 99571306 | 0.033 | 30.135 |
| rs67932684 | Feeling miserable | Frailty | G | G | 3 | 116996903 | 0.047 | 34.158 |
| rs72786276 | Feeling miserable | Frailty | A | A | 2 | 24062566 | 0.025 | 30.724 |
| rs7534703 | Feeling miserable | Frailty | T | T | 1 | 197302005 | 0.042 | 30.334 |
| rs77417259 | Feeling miserable | Frailty | A | A | 1 | 174729983 | 0.019 | 39.873 |
| rs7912226 | Feeling miserable | Frailty | A | A | 10 | 107652144 | 0.188 | 31.179 |
| rs8097041 | Feeling miserable | Frailty | T | T | 18 | 50898217 | 0.814 | 30.849 |
| rs836927 | Feeling miserable | Frailty | A | A | 3 | 107201428 | 0.041 | 33.516 |
| rs868150 | Feeling miserable | Frailty | G | G | 17 | 38213359 | 0.037 | 33.784 |
| rs10983783 | Experiencing mood swings | Frailty | T | T | 9 | 120534666 | 0.021 | 32.802 |
| rs11039149 | Experiencing mood swings | Frailty | G | G | 11 | 47276675 | 0.251 | 45.886 |
| rs11082011 | Experiencing mood swings | Frailty | T | T | 18 | 35145122 | 0.233 | 47.17 |
| rs11090039 | Experiencing mood swings | Frailty | A | A | 22 | 41496800 | 0.268 | 41.033 |
| rs11184994 | Experiencing mood swings | Frailty | T | T | 1 | 107383738 | 0.037 | 30.923 |
| rs11687833 | Experiencing mood swings | Frailty | T | T | 2 | 199499356 | 0.04 | 32.59 |
| rs12137936 | Experiencing mood swings | Frailty | G | G | 1 | 72720357 | 0.062 | 36.765 |
| rs12420205 | Experiencing mood swings | Frailty | T | T | 11 | 113394035 | 0.395 | 44.204 |
| rs1360379 | Experiencing mood swings | Frailty | T | T | 9 | 23340616 | 0.001 | 36.839 |
| rs1373921 | Experiencing mood swings | Frailty | G | G | 13 | 58634823 | 0.42 | 50.281 |
| rs1788014 | Experiencing mood swings | Frailty | G | G | 18 | 52886436 | 0.098 | 39.958 |
| rs1833070 | Experiencing mood swings | Frailty | A | A | 7 | 126123306 | 0.415 | 30.818 |
| rs1962104 | Experiencing mood swings | Frailty | C | C | 8 | 141635329 | 0.087 | 42.562 |
| rs2678897 | Experiencing mood swings | Frailty | A | A | 2 | 58169418 | 0.216 | 35.689 |
| rs28655666 | Experiencing mood swings | Frailty | A | A | 12 | 122186317 | 0.422 | 38.485 |
| rs297346 | Experiencing mood swings | Frailty | G | G | 11 | 16355771 | 0.353 | 39.07 |
| rs35789697 | Experiencing mood swings | Frailty | A | A | 2 | 144248718 | 0.433 | 35.474 |
| rs4578918 | Experiencing mood swings | Frailty | C | C | 20 | 44721656 | 0.002 | 36.914 |
| rs4651205 | Experiencing mood swings | Frailty | T | T | 1 | 174872429 | 0.089 | 30.452 |
| rs4836789 | Experiencing mood swings | Frailty | C | C | 9 | 122663466 | 0.925 | 32.705 |
| rs55657917 | Experiencing mood swings | Frailty | G | G | 17 | 43844560 | 0.001 | 86.036 |
| rs56059702 | Experiencing mood swings | Frailty | T | T | 16 | 87414635 | 0.09 | 43.452 |
| rs56318386 | Experiencing mood swings | Frailty | T | T | 9 | 11703892 | 0.127 | 37.037 |
| rs600011 | Experiencing mood swings | Frailty | C | C | 3 | 107295392 | 0.717 | 34.323 |
| rs67447472 | Experiencing mood swings | Frailty | T | T | 6 | 143062216 | 0.088 | 42.166 |
| rs68152875 | Experiencing mood swings | Frailty | A | A | 5 | 23883384 | 0.297 | 33.381 |
| rs6889822 | Experiencing mood swings | Frailty | G | G | 5 | 147846707 | 0.838 | 34.468 |
| rs6895295 | Experiencing mood swings | Frailty | T | T | 5 | 107757238 | 0.781 | 30.067 |
| rs7202252 | Experiencing mood swings | Frailty | C | C | 16 | 24741686 | 0.022 | 30.005 |
| rs784256 | Experiencing mood swings | Frailty | A | A | 18 | 53398626 | 0.012 | 48.947 |
| rs7895261 | Experiencing mood swings | Frailty | G | G | 10 | 58751054 | 0.03 | 30.722 |
| rs9344688 | Experiencing mood swings | Frailty | G | G | 6 | 64750278 | 0.948 | 33.612 |
| rs9671386 | Experiencing mood swings | Frailty | A | A | 14 | 75151047 | 0.671 | 35.79 |
| rs9929242 | Experiencing mood swings | Frailty | A | A | 16 | 74142413 | 0.023 | 31.378 |
| rs10119773 | Feeling guilty | Frailty | G | G | 9 | 23736400 | 0.02 | 46.013 |
| rs12420205 | Feeling guilty | Frailty | T | T | 11 | 113394035 | 0.607 | 36.506 |
| rs12528131 | Feeling guilty | Frailty | G | G | 6 | 105389104 | 0.089 | 41.521 |
| rs1557339 | Feeling guilty | Frailty | A | A | 18 | 35129076 | 0.441 | 40.097 |
| rs2109648 | Feeling guilty | Frailty | A | A | 17 | 64489704 | 0.383 | 34.273 |
| rs34657012 | Feeling guilty | Frailty | A | A | 2 | 23602602 | 0.11 | 35.988 |
| rs35623509 | Feeling guilty | Frailty | G | G | 9 | 120001431 | 0.172 | 31.818 |
| rs55769038 | Feeling guilty | Frailty | A | A | 11 | 13331808 | 0.003 | 47.122 |
| rs56343114 | Feeling guilty | Frailty | G | G | 2 | 23900629 | 0 | 56.524 |
| rs681875 | Feeling guilty | Frailty | A | A | 1 | 37161255 | 0.322 | 35.363 |
| rs6986 | Feeling guilty | Frailty | C | C | 6 | 30313340 | 0.235 | 52.566 |
| rs77804065 | Feeling guilty | Frailty | T | T | 17 | 43810896 | 0.147 | 38.907 |
| rs780024 | Feeling guilty | Frailty | A | A | 2 | 125052996 | 0.297 | 30.791 |
| rs10750866 | Worry too long after an embarrassing experience | Frailty | G | G | 11 | 57404779 | 0.052 | 46.77 |
| rs1983614 | Worry too long after an embarrassing experience | Frailty | T | T | 1 | 210057428 | 0.064 | 30.297 |
| rs2160515 | Worry too long after an embarrassing experience | Frailty | G | G | 12 | 16753965 | 0.006 | 39.149 |
| rs2191130 | Worry too long after an embarrassing experience | Frailty | T | T | 16 | 7667797 | 0.014 | 36.659 |
| rs34588274 | Worry too long after an embarrassing experience | Frailty | T | T | 11 | 13269946 | 0 | 69.791 |
| rs35267052 | Worry too long after an embarrassing experience | Frailty | G | G | 5 | 87949118 | 0.402 | 31.607 |
| rs35327499 | Worry too long after an embarrassing experience | Frailty | A | A | 18 | 20891631 | 0.046 | 32.382 |
| rs362307 | Worry too long after an embarrassing experience | Frailty | T | T | 4 | 3241845 | 0.584 | 37.927 |
| rs3777095 | Worry too long after an embarrassing experience | Frailty | A | A | 5 | 139660916 | 0.237 | 32.534 |
| rs3999543 | Worry too long after an embarrassing experience | Frailty | A | A | 6 | 26259807 | 0.086 | 33.121 |
| rs406204 | Worry too long after an embarrassing experience | Frailty | T | T | 5 | 113875160 | 0.102 | 29.987 |
| rs55731231 | Worry too long after an embarrassing experience | Frailty | G | G | 18 | 35183274 | 0.292 | 43.891 |
| rs6439649 | Worry too long after an embarrassing experience | Frailty | T | T | 3 | 136371691 | 0.025 | 43.349 |
| rs75022332 | Worry too long after an embarrassing experience | Frailty | T | T | 17 | 43810873 | 0.18 | 31.999 |
| rs793545 | Worry too long after an embarrassing experience | Frailty | G | G | 3 | 107091903 | 0.003 | 30.482 |
| rs7987467 | Worry too long after an embarrassing experience | Frailty | A | A | 13 | 111557399 | 0.005 | 31.696 |
| rs9811585 | Worry too long after an embarrassing experience | Frailty | G | G | 3 | 35724596 | 0.01 | 50.963 |
| rs10144845 | Feeling fed-up | Frailty | T | T | 14 | 75237770 | 0.366 | 37.462 |
| rs10409264 | Feeling fed-up | Frailty | A | A | 19 | 32896846 | 0.096 | 43.97 |
| rs10838635 | Feeling fed-up | Frailty | A | A | 11 | 46945227 | 0.386 | 40.108 |
| rs11209175 | Feeling fed-up | Frailty | T | T | 1 | 68374828 | 0.641 | 37.918 |
| rs1439253 | Feeling fed-up | Frailty | A | A | 2 | 212717215 | 0.002 | 39.247 |
| rs17018482 | Feeling fed-up | Frailty | A | A | 3 | 81129944 | 0.002 | 42.54 |
| rs17194468 | Feeling fed-up | Frailty | T | T | 2 | 204252908 | 0.022 | 33.023 |
| rs2149351 | Feeling fed-up | Frailty | G | G | 9 | 120501644 | 0.007 | 30.48 |
| rs34641928 | Feeling fed-up | Frailty | G | G | 15 | 74198181 | 0.013 | 33.695 |
| rs34668726 | Feeling fed-up | Frailty | G | G | 2 | 23895901 | 0.051 | 49.633 |
| rs4630591 | Feeling fed-up | Frailty | T | T | 17 | 44192568 | 0.001 | 74.856 |
| rs4799949 | Feeling fed-up | Frailty | T | T | 18 | 35155910 | 0.166 | 53.02 |
| rs4899292 | Feeling fed-up | Frailty | G | G | 14 | 69704052 | 0.739 | 31.506 |
| rs599550 | Feeling fed-up | Frailty | A | A | 18 | 53252388 | 0.007 | 58.254 |
| rs6031966 | Feeling fed-up | Frailty | T | T | 20 | 43692633 | 0.016 | 32.569 |
| rs61744010 | Feeling fed-up | Frailty | T | T | 16 | 88776499 | 0.251 | 30.351 |
| rs6862766 | Feeling fed-up | Frailty | T | T | 5 | 24156932 | 0.095 | 38.222 |
| rs7334060 | Feeling fed-up | Frailty | C | C | 13 | 55728751 | 0.055 | 29.954 |
| rs77087420 | Feeling fed-up | Frailty | G | G | 4 | 123122856 | 0.344 | 31.202 |
| rs783537 | Feeling fed-up | Frailty | G | G | 15 | 83252807 | 0.053 | 34.909 |
| rs7912226 | Feeling fed-up | Frailty | A | A | 10 | 107652144 | 0.065 | 42.583 |
| rs9842349 | Feeling fed-up | Frailty | T | T | 3 | 45320986 | 0.021 | 32.248 |
| rs997091 | Feeling fed-up | Frailty | C | C | 4 | 28692943 | 0.78 | 34.412 |
| rs10501320 | Feeling nervous | Frailty | C | C | 11 | 47293799 | 0.5 | 37.364 |
| rs10935184 | Feeling nervous | Frailty | C | C | 3 | 136153468 | 0.083 | 34.31 |
| rs11030107 | Feeling nervous | Frailty | G | G | 11 | 27694835 | 0.049 | 35.135 |
| rs11619066 | Feeling nervous | Frailty | C | C | 13 | 59141522 | 0.788 | 32.478 |
| rs117618307 | Feeling nervous | Frailty | G | G | 15 | 42030849 | 0.003 | 32.335 |
| rs12729445 | Feeling nervous | Frailty | T | T | 1 | 225558489 | 0.012 | 35.397 |
| rs13395141 | Feeling nervous | Frailty | T | T | 2 | 148955901 | 0.132 | 30.235 |
| rs1368549 | Feeling nervous | Frailty | T | T | 2 | 104064726 | 0 | 55.635 |
| rs1375545 | Feeling nervous | Frailty | T | T | 3 | 85604320 | 0 | 63.107 |
| rs1427041 | Feeling nervous | Frailty | G | G | 18 | 35208320 | 0.163 | 34.354 |
| rs17884466 | Feeling nervous | Frailty | G | G | 17 | 29546765 | 0.039 | 30.975 |
| rs2205129 | Feeling nervous | Frailty | A | A | 21 | 41464434 | 0.082 | 32.633 |
| rs2407746 | Feeling nervous | Frailty | G | G | 8 | 4937757 | 0.01 | 35.734 |
| rs2853779 | Feeling nervous | Frailty | A | A | 14 | 75368508 | 0.425 | 48.225 |
| rs2930456 | Feeling nervous | Frailty | C | C | 10 | 107050168 | 0.012 | 30.572 |
| rs332828 | Feeling nervous | Frailty | A | A | 1 | 61742693 | 0.041 | 36.177 |
| rs4129585 | Feeling nervous | Frailty | C | C | 8 | 143312933 | 0.099 | 41.111 |
| rs4787491 | Feeling nervous | Frailty | G | G | 16 | 30015337 | 0.057 | 34.793 |
| rs4919695 | Feeling nervous | Frailty | G | G | 10 | 104704017 | 0 | 55.272 |
| rs5758209 | Feeling nervous | Frailty | G | G | 22 | 41461865 | 0.002 | 31.674 |
| rs62062288 | Feeling nervous | Frailty | A | A | 17 | 44096553 | 0.009 | 65.338 |
| rs62212171 | Feeling nervous | Frailty | C | C | 20 | 32987687 | 0.005 | 39.999 |
| rs6919397 | Feeling nervous | Frailty | G | G | 6 | 126659043 | 0.039 | 35.641 |
| rs6948180 | Feeling nervous | Frailty | A | A | 7 | 97374775 | 0.013 | 37.407 |
| rs7026674 | Feeling nervous | Frailty | T | T | 9 | 126452936 | 0.329 | 32.6 |
| rs707916 | Feeling nervous | Frailty | A | A | 6 | 31697558 | 0.021 | 54.131 |
| rs7792856 | Feeling nervous | Frailty | C | C | 7 | 82066457 | 0.016 | 32.074 |
| rs8050237 | Feeling nervous | Frailty | G | G | 16 | 87492449 | 0.01 | 30.688 |
| rs9688806 | Feeling nervous | Frailty | T | T | 6 | 98630881 | 0.225 | 40.876 |
| rs974711 | Feeling nervous | Frailty | A | A | 4 | 90737327 | 0.316 | 36.949 |
| rs9835772 | Feeling nervous | Frailty | T | T | 3 | 85766025 | 0.001 | 38.142 |
| rs9879090 | Feeling nervous | Frailty | C | C | 3 | 52648265 | 0.01 | 36.472 |
| rs10034259 | Feeling worry | Frailty | C | C | 4 | 104933753 | 0.02 | 31.039 |
| rs10096972 | Feeling worry | Frailty | C | C | 8 | 10289988 | 0.017 | 34.848 |
| rs10750486 | Feeling worry | Frailty | A | A | 11 | 131463916 | 0.002 | 31.901 |
| rs10765762 | Feeling worry | Frailty | T | T | 11 | 95434061 | 0.003 | 36.604 |
| rs10959797 | Feeling worry | Frailty | A | A | 9 | 11431990 | 0.248 | 31.374 |
| rs11152363 | Feeling worry | Frailty | A | A | 18 | 53057188 | 0.072 | 33.174 |
| rs116962250 | Feeling worry | Frailty | A | A | 19 | 743362 | 0.462 | 34.227 |
| rs13054099 | Feeling worry | Frailty | C | C | 22 | 41215672 | 0.52 | 32.175 |
| rs1375311 | Feeling worry | Frailty | A | A | 2 | 168621005 | 0.209 | 33.383 |
| rs1542212 | Feeling worry | Frailty | G | G | 3 | 35683935 | 0.073 | 33.901 |
| rs167915 | Feeling worry | Frailty | T | T | 11 | 133797581 | 0.291 | 34.972 |
| rs1697692 | Feeling worry | Frailty | T | T | 3 | 107307503 | 0.004 | 31.751 |
| rs17688916 | Feeling worry | Frailty | A | A | 17 | 43778680 | 0.061 | 46.627 |
| rs187580 | Feeling worry | Frailty | G | G | 5 | 102627355 | 0.227 | 40.934 |
| rs2367724 | Feeling worry | Frailty | T | T | 1 | 44107428 | 0.002 | 35.507 |
| rs2389499 | Feeling worry | Frailty | G | G | 4 | 118969887 | 0.029 | 30.397 |
| rs2488398 | Feeling worry | Frailty | C | C | 1 | 197701376 | 0.009 | 51.591 |
| rs274632 | Feeling worry | Frailty | A | A | 7 | 86269181 | 0.207 | 30.417 |
| rs28510415 | Feeling worry | Frailty | G | G | 9 | 98245026 | 0.051 | 30.748 |
| rs3026389 | Feeling worry | Frailty | G | G | 11 | 31813529 | 0.057 | 35.166 |
| rs3808072 | Feeling worry | Frailty | T | T | 7 | 127595573 | 0.012 | 41.487 |
| rs4396680 | Feeling worry | Frailty | G | G | 2 | 10178236 | 0.121 | 32.107 |
| rs4659554 | Feeling worry | Frailty | G | G | 1 | 240077309 | 0.128 | 30.347 |
| rs4919695 | Feeling worry | Frailty | G | G | 10 | 104704017 | 0 | 54.158 |
| rs55997507 | Feeling worry | Frailty | C | C | 16 | 7666664 | 0.089 | 42.799 |
| rs57462170 | Feeling worry | Frailty | A | A | 3 | 50239803 | 0.081 | 36.499 |
| rs58084604 | Feeling worry | Frailty | T | T | 18 | 57849429 | 0.004 | 38.029 |
| rs61957597 | Feeling worry | Frailty | G | G | 13 | 58983958 | 0.242 | 37.718 |
| rs62250713 | Feeling worry | Frailty | G | G | 3 | 85513793 | 0 | 71.036 |
| rs6791142 | Feeling worry | Frailty | C | C | 3 | 136377377 | 0.044 | 36.246 |
| rs6798941 | Feeling worry | Frailty | T | T | 3 | 52893465 | 0 | 58.484 |
| rs72893199 | Feeling worry | Frailty | C | C | 18 | 35182708 | 0.284 | 42.919 |
| rs7543687 | Feeling worry | Frailty | T | T | 1 | 21657507 | 0.061 | 32.426 |
| rs7567451 | Feeling worry | Frailty | T | T | 2 | 157053380 | 0.329 | 30.558 |
| rs9462364 | Feeling worry | Frailty | G | G | 6 | 11997050 | 0.003 | 35.504 |
| rs10117184 | Feeling hurt | Frailty | G | G | 9 | 11256918 | 0.198 | 33.985 |
| rs10210652 | Feeling hurt | Frailty | A | A | 2 | 148469593 | 0.125 | 39.733 |
| rs10511285 | Feeling hurt | Frailty | G | G | 3 | 110103126 | 0.08 | 31.631 |
| rs10891564 | Feeling hurt | Frailty | A | A | 11 | 113374013 | 0.239 | 38.975 |
| rs11663050 | Feeling hurt | Frailty | G | G | 18 | 35201153 | 0.016 | 66.342 |
| rs11767715 | Feeling hurt | Frailty | T | T | 7 | 39129941 | 0.205 | 34.269 |
| rs12028465 | Feeling hurt | Frailty | A | A | 1 | 53736136 | 0.265 | 33.342 |
| rs1231375 | Feeling hurt | Frailty | C | C | 9 | 25755952 | 0.002 | 32.556 |
| rs12933611 | Feeling hurt | Frailty | G | G | 16 | 13765383 | 0.003 | 30.124 |
| rs1389993 | Feeling hurt | Frailty | C | C | 21 | 19032949 | 0.655 | 35.002 |
| rs145965565 | Feeling hurt | Frailty | G | G | 9 | 98273305 | 0.046 | 44.609 |
| rs17532098 | Feeling hurt | Frailty | T | T | 9 | 96262725 | 0.985 | 31.127 |
| rs2027798 | Feeling hurt | Frailty | T | T | 5 | 149439044 | 0.022 | 32.706 |
| rs2102923 | Feeling hurt | Frailty | G | G | 14 | 69662105 | 0.65 | 33.765 |
| rs219226 | Feeling hurt | Frailty | C | C | 2 | 205014049 | 0.038 | 30.02 |
| rs2488401 | Feeling hurt | Frailty | T | T | 1 | 197702401 | 0.055 | 42.81 |
| rs4652676 | Feeling hurt | Frailty | A | A | 1 | 181698693 | 0.077 | 30.339 |
| rs4702 | Feeling hurt | Frailty | A | A | 15 | 91426560 | 0.002 | 36.006 |
| rs4791774 | Feeling hurt | Frailty | G | G | 17 | 8932119 | 0.004 | 30.542 |
| rs4868774 | Feeling hurt | Frailty | G | G | 5 | 166054544 | 0.09 | 32.37 |
| rs545853 | Feeling hurt | Frailty | A | A | 18 | 77529844 | 0.006 | 31.586 |
| rs55657917 | Feeling hurt | Frailty | G | G | 17 | 43844560 | 0 | 125.78 |
| rs62035176 | Feeling hurt | Frailty | A | A | 16 | 82878155 | 0.484 | 30.749 |
| rs62268962 | Feeling hurt | Frailty | T | T | 3 | 113917372 | 0.009 | 30.946 |
| rs73480560 | Feeling hurt | Frailty | T | T | 11 | 57401400 | 0.134 | 32.33 |
| rs10218528 | Feeling tense | Frailty | A | A | 1 | 174447588 | 0.353 | 31.104 |
| rs10767733 | Feeling tense | Frailty | A | A | 11 | 28642320 | 0.377 | 39.976 |
| rs11090045 | Feeling tense | Frailty | A | A | 22 | 41753603 | 0.1 | 42.373 |
| rs1147851 | Feeling tense | Frailty | G | G | 6 | 147983560 | 0.149 | 34.506 |
| rs1450832 | Feeling tense | Frailty | A | A | 7 | 113863384 | 0.103 | 37.509 |
| rs2097247 | Feeling tense | Frailty | T | T | 3 | 50290902 | 0.101 | 35.27 |
| rs2100888 | Feeling tense | Frailty | A | A | 1 | 8659282 | 0.011 | 37.983 |
| rs28738966 | Feeling tense | Frailty | A | A | 5 | 87248375 | 0.021 | 38.851 |
| rs3740393 | Feeling tense | Frailty | C | C | 10 | 104636655 | 0.014 | 36.163 |
| rs3751855 | Feeling tense | Frailty | C | C | 16 | 31091209 | 0.1 | 31.871 |
| rs4129585 | Feeling tense | Frailty | C | C | 8 | 143312933 | 0.132 | 37.829 |
| rs4671330 | Feeling tense | Frailty | A | A | 2 | 59042847 | 0.005 | 38.088 |
| rs6883228 | Feeling tense | Frailty | C | C | 5 | 153669155 | 0.04 | 30.366 |
| rs6891955 | Feeling tense | Frailty | C | C | 5 | 64838758 | 0.01 | 35.907 |
| rs7194615 | Feeling tense | Frailty | C | C | 16 | 82769498 | 0.051 | 31.772 |
| rs78379741 | Feeling tense | Frailty | C | C | 20 | 18685835 | 0.003 | 37.925 |
| rs79861172 | Feeling tense | Frailty | A | A | 2 | 148607438 | 0.038 | 29.908 |
| rs9527336 | Feeling tense | Frailty | A | A | 13 | 55783042 | 0.028 | 34.775 |
| rs9811546 | Feeling tense | Frailty | A | A | 3 | 85391672 | 0.024 | 34.005 |

**Table S3** The effects of frailty on neuroticism-related phenotype.

| **Exposure** | **Outcome** | **Method** | **N of SNP** | **β (95%CI)** | **se** | ***P*** | **OR (95%CI)** | ***P _fdr_*** |
| --- | --- | --- | --- | --- | --- | --- | --- | --- |
| Frailty | Neuroticism | IVW | 12 | 0.239(0.160-0.319) | 0.041 | < 0.001 | 1.270(1.173-1.375) | < 0.001 |
| Frailty | Irritable mood | IVW | 13 | 0.155(0.068-0.242) | 0.044 | < 0.001 | 1.167(1.07-1.273) | 0.001 |
| Frailty | Feeling lonely | IVW | 14 | 0.198(0.132-0.264) | 0.034 | < 0.001 | 1.219(1.141-1.303) | < 0.001 |
| Frailty | Feeling miserable | IVW | 12 | 0.197(0.129-0.264) | 0.034 | < 0.001 | 1.217(1.138-1.302) | < 0.001 |
| Frailty | Experiencing mood swings | IVW | 11 | 0.166(0.102-0.23) | 0.033 | < 0.001 | 1.181(1.107-1.259) | < 0.001 |
| Frailty | Feeling guilty | IVW | 12 | 0.077(-0.015-0.168) | 0.047 | 0.099 | 1.080(0.986-1.183) | 0.099 |
| Frailty | Worry too long after an embarrassing experience | IVW | 13 | 0.113(0.007-0.219) | 0.054 | 0.037 | 1.120(1.007-1.245) | 0.041 |
| Frailty | Feeling fed-up | IVW | 14 | 0.249(0.168-0.331) | 0.042 | < 0.001 | 1.283(1.183-1.393) | < 0.001 |
| Frailty | Feeling nervous | IVW | 14 | 0.135(0.036-0.234) | 0.05 | 0.007 | 1.144(1.037-1.263) | 0.009 |
| Frailty | Feeling worry | IVW | 13 | 0.174(0.1-0.248) | 0.038 | < 0.001 | 1.19(1.105-1.282) | < 0.001 |
| Frailty | Feeling hurt | IVW | 13 | 0.119(0.036-0.202) | 0.042 | 0.005 | 1.126(1.037-1.224) | 0.007 |
| Frailty | Feeling tense | IVW | 12 | 0.125(0.04-0.21) | 0.043 | 0.004 | 1.133(1.041-1.234) | 0.006 |
| Frailty | Neuroticism | MR Egger | 12 | 0.119(-0.226-0.464) | 0.176 | 0.514 | 1.126(0.798-1.590) | 0.881 |
| Frailty | Irritable mood | MR Egger | 13 | -0.071(-0.434-0.292) | 0.185 | 0.708 | 0.931(0.648-1.339) | 0.944 |
| Frailty | Feeling lonely | MR Egger | 14 | -0.109(-0.366-0.147) | 0.131 | 0.419 | 0.896(0.694-1.158) | 0.838 |
| Frailty | Feeling miserable | MR Egger | 12 | 0.152(-0.15-0.453) | 0.154 | 0.348 | 1.164(0.861-1.573) | 0.835 |
| Frailty | Experiencing mood swings | MR Egger | 11 | 0.155(-0.112-0.423) | 0.137 | 0.285 | 1.168(0.894-1.526) | 0.835 |
| Frailty | Feeling guilty | MR Egger | 12 | 0.031(-0.374-0.437) | 0.207 | 0.883 | 1.032(0.688-1.547) | 0.962 |
| Frailty | Worry too long after an embarrassing experience | MR Egger | 13 | -0.041(-0.504-0.421) | 0.236 | 0.864 | 0.960(0.604-1.524) | 0.962 |
| Frailty | Feeling fed-up | MR Egger | 14 | 0.103(-0.262-0.469) | 0.186 | 0.589 | 1.109(0.77-1.598) | 0.884 |
| Frailty | Feeling nervous | MR Egger | 14 | 0.273(-0.173-0.718) | 0.227 | 0.254 | 1.313(0.841-2.051) | 0.835 |
| Frailty | Feeling worry | MR Egger | 13 | 0.291(-0.038-0.619) | 0.168 | 0.111 | 1.337(0.963-1.858) | 0.666 |
| Frailty | Feeling hurt | MR Egger | 13 | 0.356(0.008-0.704) | 0.178 | 0.070 | 1.428(1.008-2.023) | 0.666 |
| Frailty | Feeling tense | MR Egger | 12 | -0.009(-0.375-0.357) | 0.187 | 0.962 | 0.991(0.687-1.428) | 0.962 |
| Frailty | Neuroticism | Weighted median | 12 | 0.232(0.139-0.325) | 0.048 | < 0.001 | 1.261(1.149-1.385) | < 0.001 |
| Frailty | Irritable mood | Weighted median | 13 | 0.089(-0.008-0.185) | 0.049 | 0.071 | 1.093(0.992-1.203) | 0.077 |
| Frailty | Feeling lonely | Weighted median | 14 | 0.184(0.097-0.271) | 0.044 | < 0.001 | 1.202(1.102-1.312) | < 0.001 |
| Frailty | Feeling miserable | Weighted median | 12 | 0.167(0.078-0.256) | 0.045 | < 0.001 | 1.181(1.081-1.291) | < 0.001 |
| Frailty | Experiencing mood swings | Weighted median | 11 | 0.168(0.078-0.258) | 0.046 | < 0.001 | 1.183(1.081-1.295) | < 0.001 |
| Frailty | Feeling guilty | Weighted median | 12 | 0.116(0.025-0.207) | 0.046 | 0.013 | 1.123(1.025-1.230) | 0.019 |
| Frailty | Worry too long after an embarrassing experience | Weighted median | 13 | 0.047(-0.045-0.14) | 0.047 | 0.315 | 1.049(0.956-1.150) | 0.315 |
| Frailty | Feeling fed-up | Weighted median | 14 | 0.182(0.088-0.275) | 0.048 | < 0.001 | 1.199(1.093-1.316) | < 0.001 |
| Frailty | Feeling nervous | Weighted median | 14 | 0.172(0.082-0.261) | 0.046 | < 0.001 | 1.187(1.086-1.299) | < 0.001 |
| Frailty | Feeling worry | Weighted median | 13 | 0.201(0.115-0.287) | 0.044 | < 0.001 | 1.223(1.122-1.332) | < 0.001 |
| Frailty | Feeling hurt | Weighted median | 13 | 0.105(0.008-0.201) | 0.049 | 0.033 | 1.111(1.009-1.223) | 0.040 |
| Frailty | Feeling tense | Weighted median | 12 | 0.107(0.016-0.198) | 0.047 | 0.022 | 1.113(1.016-1.219) | 0.029 |
| Frailty | Neuroticism | Weighted mode | 12 | 0.259(0.126-0.392) | 0.068 | 0.003 | 1.295(1.134-1.480) | 0.012 |
| Frailty | Irritable mood | Weighted mode | 13 | 0.062(-0.057-0.18) | 0.06 | 0.328 | 1.063(0.945-1.197) | 0.338 |
| Frailty | Feeling lonely | Weighted mode | 14 | 0.234(0.113-0.356) | 0.062 | 0.002 | 1.264(1.12-1.427) | 0.012 |
| Frailty | Feeling miserable | Weighted mode | 12 | 0.165(0.042-0.288) | 0.063 | 0.023 | 1.180(1.043-1.334) | 0.053 |
| Frailty | Experiencing mood swings | Weighted mode | 11 | 0.156(0.037-0.274) | 0.061 | 0.028 | 1.168(1.037-1.316) | 0.053 |
| Frailty | Feeling guilty | Weighted mode | 12 | 0.135(0.023-0.248) | 0.057 | 0.038 | 1.145(1.023-1.281) | 0.057 |
| Frailty | Worry too long after an embarrassing experience | Weighted mode | 13 | 0.055(-0.053-0.164) | 0.055 | 0.338 | 1.057(0.948-1.178) | 0.338 |
| Frailty | Feeling fed-up | Weighted mode | 14 | 0.147(0.028-0.267) | 0.061 | 0.031 | 1.159(1.029-1.306) | 0.053 |
| Frailty | Feeling nervous | Weighted mode | 14 | 0.173(0.055-0.29) | 0.06 | 0.013 | 1.188(1.056-1.337) | 0.039 |
| Frailty | Feeling worry | Weighted mode | 13 | 0.229(0.111-0.348) | 0.06 | 0.003 | 1.258(1.117-1.416) | 0.012 |
| Frailty | Feeling hurt | Weighted mode | 13 | 0.149(0.012-0.285) | 0.07 | 0.054 | 1.16(1.012-1.330) | 0.073 |
| Frailty | Feeling tense | Weighted mode | 12 | 0.088(-0.032-0.207) | 0.061 | 0.179 | 1.091(0.969-1.230) | 0.215 |

**Table S4** Characteristics of genetic variants used to estimate the effect of frailty on neuroticism-related phenotype.

| **SNP** | **Exposure** | **Outcome** | **Allele of exposure** | **Allele of outcome** | **chr** | **pos** | **Steiger *P*** | **F** |
| --- | --- | --- | --- | --- | --- | --- | --- | --- |
| rs12739243 | Frailty | Neuroticism | C | C | 1 | 210302043 | <0.001 | 35.295 |
| rs1363103 | Frailty | Neuroticism | C | C | 5 | 103917837 | 0.001 | 30.126 |
| rs17612102 | Frailty | Neuroticism | C | C | 15 | 52264094 | <0.001 | 29.575 |
| rs2071207 | Frailty | Neuroticism | C | C | 3 | 50159844 | 0.005 | 30.583 |
| rs2396766 | Frailty | Neuroticism | A | A | 7 | 114318071 | 0.001 | 35.296 |
| rs3959554 | Frailty | Neuroticism | G | G | 15 | 41443924 | 0.008 | 30.453 |
| rs4146140 | Frailty | Neuroticism | T | T | 10 | 61885362 | <0.001 | 32.411 |
| rs4952693 | Frailty | Neuroticism | T | T | 2 | 44151808 | 0.001 | 30.865 |
| rs56299474 | Frailty | Neuroticism | A | A | 8 | 21992804 | <0.001 | 29.166 |
| rs583514 | Frailty | Neuroticism | C | C | 3 | 173114167 | 0.001 | 34.685 |
| rs82334 | Frailty | Neuroticism | C | C | 4 | 3225371 | <0.001 | 37.785 |
| rs9275160 | Frailty | Neuroticism | A | A | 6 | 32652620 | <0.001 | 114.781 |
| rs10891490 | Frailty | Irritable mood | C | C | 11 | 112885527 | 0.007 | 29.934 |
| rs12739243 | Frailty | Irritable mood | C | C | 1 | 210302043 | <0.001 | 35.295 |
| rs1363103 | Frailty | Irritable mood | C | C | 5 | 103917837 | 0.003 | 30.126 |
| rs17612102 | Frailty | Irritable mood | C | C | 15 | 52264094 | <0.001 | 29.575 |
| rs2071207 | Frailty | Irritable mood | C | C | 3 | 50159844 | <0.001 | 30.583 |
| rs3959554 | Frailty | Irritable mood | G | G | 15 | 41443924 | <0.001 | 30.453 |
| rs4146140 | Frailty | Irritable mood | T | T | 10 | 61885362 | <0.001 | 32.411 |
| rs4952693 | Frailty | Irritable mood | T | T | 2 | 44151808 | <0.001 | 30.865 |
| rs56299474 | Frailty | Irritable mood | A | A | 8 | 21992804 | 0.005 | 29.166 |
| rs583514 | Frailty | Irritable mood | C | C | 3 | 173114167 | <0.001 | 34.685 |
| rs8089807 | Frailty | Irritable mood | T | T | 18 | 39322639 | 0.002 | 32.721 |
| rs82334 | Frailty | Irritable mood | C | C | 4 | 3225371 | <0.001 | 37.785 |
| rs9275160 | Frailty | Irritable mood | A | A | 6 | 32652620 | <0.001 | 114.781 |
| rs10891490 | Frailty | Feeling lonely | C | C | 11 | 112885527 | 0.007 | 29.934 |
| rs12739243 | Frailty | Feeling lonely | C | C | 1 | 210302043 | <0.001 | 35.295 |
| rs1363103 | Frailty | Feeling lonely | C | C | 5 | 103917837 | 0.001 | 30.126 |
| rs17612102 | Frailty | Feeling lonely | C | C | 15 | 52264094 | 0.001 | 29.575 |
| rs2071207 | Frailty | Feeling lonely | C | C | 3 | 50159844 | <0.001 | 30.583 |
| rs2396766 | Frailty | Feeling lonely | A | A | 7 | 114318071 | 0.003 | 35.296 |
| rs3959554 | Frailty | Feeling lonely | G | G | 15 | 41443924 | 0.001 | 30.453 |
| rs4146140 | Frailty | Feeling lonely | T | T | 10 | 61885362 | <0.001 | 32.411 |
| rs4952693 | Frailty | Feeling lonely | T | T | 2 | 44151808 | <0.001 | 30.865 |
| rs56299474 | Frailty | Feeling lonely | A | A | 8 | 21992804 | <0.001 | 29.166 |
| rs583514 | Frailty | Feeling lonely | C | C | 3 | 173114167 | <0.001 | 34.685 |
| rs8089807 | Frailty | Feeling lonely | T | T | 18 | 39322639 | 0.001 | 32.721 |
| rs82334 | Frailty | Feeling lonely | C | C | 4 | 3225371 | <0.001 | 37.785 |
| rs9275160 | Frailty | Feeling lonely | A | A | 6 | 32652620 | <0.001 | 114.781 |
| rs12739243 | Frailty | Feeling miserable | C | C | 1 | 210302043 | <0.001 | 35.295 |
| rs1363103 | Frailty | Feeling miserable | C | C | 5 | 103917837 | <0.001 | 30.126 |
| rs17612102 | Frailty | Feeling miserable | C | C | 15 | 52264094 | <0.001 | 29.575 |
| rs2396766 | Frailty | Feeling miserable | A | A | 7 | 114318071 | <0.001 | 35.296 |
| rs3959554 | Frailty | Feeling miserable | G | G | 15 | 41443924 | 0.002 | 30.453 |
| rs4146140 | Frailty | Feeling miserable | T | T | 10 | 61885362 | <0.001 | 32.411 |
| rs4952693 | Frailty | Feeling miserable | T | T | 2 | 44151808 | 0.002 | 30.865 |
| rs56299474 | Frailty | Feeling miserable | A | A | 8 | 21992804 | <0.001 | 29.166 |
| rs583514 | Frailty | Feeling miserable | C | C | 3 | 173114167 | <0.001 | 34.685 |
| rs8089807 | Frailty | Feeling miserable | T | T | 18 | 39322639 | 0.005 | 32.721 |
| rs82334 | Frailty | Feeling miserable | C | C | 4 | 3225371 | <0.001 | 37.785 |
| rs9275160 | Frailty | Feeling miserable | A | A | 6 | 32652620 | <0.001 | 114.781 |
| rs12739243 | Frailty | Experiencing mood swings | C | C | 1 | 210302043 | <0.001 | 35.295 |
| rs1363103 | Frailty | Experiencing mood swings | C | C | 5 | 103917837 | 0.002 | 30.126 |
| rs17612102 | Frailty | Experiencing mood swings | C | C | 15 | 52264094 | <0.001 | 29.575 |
| rs2396766 | Frailty | Experiencing mood swings | A | A | 7 | 114318071 | <0.001 | 35.296 |
| rs3959554 | Frailty | Experiencing mood swings | G | G | 15 | 41443924 | 0.001 | 30.453 |
| rs4146140 | Frailty | Experiencing mood swings | T | T | 10 | 61885362 | <0.001 | 32.411 |
| rs4952693 | Frailty | Experiencing mood swings | T | T | 2 | 44151808 | <0.001 | 30.865 |
| rs56299474 | Frailty | Experiencing mood swings | A | A | 8 | 21992804 | <0.001 | 29.166 |
| rs583514 | Frailty | Experiencing mood swings | C | C | 3 | 173114167 | <0.001 | 34.685 |
| rs82334 | Frailty | Experiencing mood swings | C | C | 4 | 3225371 | <0.001 | 37.785 |
| rs9275160 | Frailty | Experiencing mood swings | A | A | 6 | 32652620 | <0.001 | 114.781 |
| rs12739243 | Frailty | Feeling guilty | C | C | 1 | 210302043 | <0.001 | 35.295 |
| rs1363103 | Frailty | Feeling guilty | C | C | 5 | 103917837 | <0.001 | 30.126 |
| rs17612102 | Frailty | Feeling guilty | C | C | 15 | 52264094 | <0.001 | 29.575 |
| rs2071207 | Frailty | Feeling guilty | C | C | 3 | 50159844 | <0.001 | 30.583 |
| rs2396766 | Frailty | Feeling guilty | A | A | 7 | 114318071 | <0.001 | 35.296 |
| rs3959554 | Frailty | Feeling guilty | G | G | 15 | 41443924 | <0.001 | 30.453 |
| rs4146140 | Frailty | Feeling guilty | T | T | 10 | 61885362 | <0.001 | 32.411 |
| rs4952693 | Frailty | Feeling guilty | T | T | 2 | 44151808 | <0.001 | 30.865 |
| rs56299474 | Frailty | Feeling guilty | A | A | 8 | 21992804 | <0.001 | 29.166 |
| rs583514 | Frailty | Feeling guilty | C | C | 3 | 173114167 | <0.001 | 34.685 |
| rs82334 | Frailty | Feeling guilty | C | C | 4 | 3225371 | <0.001 | 37.785 |
| rs9275160 | Frailty | Feeling guilty | A | A | 6 | 32652620 | <0.001 | 114.781 |
| rs10891490 | Frailty | Worry too long after an embarrassing experience | C | C | 11 | 112885527 | 0.006 | 29.934 |
| rs12739243 | Frailty | Worry too long after an embarrassing experience | C | C | 1 | 210302043 | <0.001 | 35.295 |
| rs1363103 | Frailty | Worry too long after an embarrassing experience | C | C | 5 | 103917837 | <0.001 | 30.126 |
| rs17612102 | Frailty | Worry too long after an embarrassing experience | C | C | 15 | 52264094 | <0.001 | 29.575 |
| rs2071207 | Frailty | Worry too long after an embarrassing experience | C | C | 3 | 50159844 | 0.001 | 30.583 |
| rs2396766 | Frailty | Worry too long after an embarrassing experience | A | A | 7 | 114318071 | <0.001 | 35.296 |
| rs3959554 | Frailty | Worry too long after an embarrassing experience | G | G | 15 | 41443924 | <0.001 | 30.453 |
| rs4146140 | Frailty | Worry too long after an embarrassing experience | T | T | 10 | 61885362 | <0.001 | 32.411 |
| rs4952693 | Frailty | Worry too long after an embarrassing experience | T | T | 2 | 44151808 | 0.006 | 30.865 |
| rs56299474 | Frailty | Worry too long after an embarrassing experience | A | A | 8 | 21992804 | <0.001 | 29.166 |
| rs583514 | Frailty | Worry too long after an embarrassing experience | C | C | 3 | 173114167 | <0.001 | 34.685 |
| rs8089807 | Frailty | Worry too long after an embarrassing experience | T | T | 18 | 39322639 | 0.004 | 32.721 |
| rs9275160 | Frailty | Worry too long after an embarrassing experience | A | A | 6 | 32652620 | <0.001 | 114.781 |
| rs10891490 | Frailty | Feeling fed-up | C | C | 11 | 112885527 | 0.011 | 29.934 |
| rs12739243 | Frailty | Feeling fed-up | C | C | 1 | 210302043 | <0.001 | 35.295 |
| rs1363103 | Frailty | Feeling fed-up | C | C | 5 | 103917837 | 0.001 | 30.126 |
| rs17612102 | Frailty | Feeling fed-up | C | C | 15 | 52264094 | <0.001 | 29.575 |
| rs2071207 | Frailty | Feeling fed-up | C | C | 3 | 50159844 | 0.014 | 30.583 |
| rs2396766 | Frailty | Feeling fed-up | A | A | 7 | 114318071 | <0.001 | 35.296 |
| rs3959554 | Frailty | Feeling fed-up | G | G | 15 | 41443924 | 0.015 | 30.453 |
| rs4146140 | Frailty | Feeling fed-up | T | T | 10 | 61885362 | <0.001 | 32.411 |
| rs4952693 | Frailty | Feeling fed-up | T | T | 2 | 44151808 | <0.001 | 30.865 |
| rs56299474 | Frailty | Feeling fed-up | A | A | 8 | 21992804 | <0.001 | 29.166 |
| rs583514 | Frailty | Feeling fed-up | C | C | 3 | 173114167 | 0.002 | 34.685 |
| rs8089807 | Frailty | Feeling fed-up | T | T | 18 | 39322639 | 0.01 | 32.721 |
| rs82334 | Frailty | Feeling fed-up | C | C | 4 | 3225371 | <0.001 | 37.785 |
| rs9275160 | Frailty | Feeling fed-up | A | A | 6 | 32652620 | <0.001 | 114.781 |
| rs10891490 | Frailty | Feeling nervous | C | C | 11 | 112885527 | 0.023 | 29.934 |
| rs12739243 | Frailty | Feeling nervous | C | C | 1 | 210302043 | <0.001 | 35.295 |
| rs1363103 | Frailty | Feeling nervous | C | C | 5 | 103917837 | <0.001 | 30.126 |
| rs17612102 | Frailty | Feeling nervous | C | C | 15 | 52264094 | <0.001 | 29.575 |
| rs2071207 | Frailty | Feeling nervous | C | C | 3 | 50159844 | <0.001 | 30.583 |
| rs2396766 | Frailty | Feeling nervous | A | A | 7 | 114318071 | <0.001 | 35.296 |
| rs3959554 | Frailty | Feeling nervous | G | G | 15 | 41443924 | <0.001 | 30.453 |
| rs4146140 | Frailty | Feeling nervous | T | T | 10 | 61885362 | <0.001 | 32.411 |
| rs4952693 | Frailty | Feeling nervous | T | T | 2 | 44151808 | <0.001 | 30.865 |
| rs56299474 | Frailty | Feeling nervous | A | A | 8 | 21992804 | <0.001 | 29.166 |
| rs583514 | Frailty | Feeling nervous | C | C | 3 | 173114167 | <0.001 | 34.685 |
| rs8089807 | Frailty | Feeling nervous | T | T | 18 | 39322639 | 0.01 | 32.721 |
| rs82334 | Frailty | Feeling nervous | C | C | 4 | 3225371 | <0.001 | 37.785 |
| rs9275160 | Frailty | Feeling nervous | A | A | 6 | 32652620 | <0.001 | 114.781 |
| rs12739243 | Frailty | Feeling worry | C | C | 1 | 210302043 | <0.001 | 35.295 |
| rs1363103 | Frailty | Feeling worry | C | C | 5 | 103917837 | <0.001 | 30.126 |
| rs17612102 | Frailty | Feeling worry | C | C | 15 | 52264094 | <0.001 | 29.575 |
| rs2071207 | Frailty | Feeling worry | C | C | 3 | 50159844 | 0.001 | 30.583 |
| rs2396766 | Frailty | Feeling worry | A | A | 7 | 114318071 | <0.001 | 35.296 |
| rs3959554 | Frailty | Feeling worry | G | G | 15 | 41443924 | 0.001 | 30.453 |
| rs4146140 | Frailty | Feeling worry | T | T | 10 | 61885362 | <0.001 | 32.411 |
| rs4952693 | Frailty | Feeling worry | T | T | 2 | 44151808 | <0.001 | 30.865 |
| rs56299474 | Frailty | Feeling worry | A | A | 8 | 21992804 | <0.001 | 29.166 |
| rs583514 | Frailty | Feeling worry | C | C | 3 | 173114167 | <0.001 | 34.685 |
| rs8089807 | Frailty | Feeling worry | T | T | 18 | 39322639 | 0.003 | 32.721 |
| rs82334 | Frailty | Feeling worry | C | C | 4 | 3225371 | <0.001 | 37.785 |
| rs9275160 | Frailty | Feeling worry | A | A | 6 | 32652620 | <0.001 | 114.781 |
| rs12739243 | Frailty | Feeling hurt | C | C | 1 | 210302043 | <0.001 | 35.295 |
| rs1363103 | Frailty | Feeling hurt | C | C | 5 | 103917837 | <0.001 | 30.126 |
| rs17612102 | Frailty | Feeling hurt | C | C | 15 | 52264094 | <0.001 | 29.575 |
| rs2071207 | Frailty | Feeling hurt | C | C | 3 | 50159844 | <0.001 | 30.583 |
| rs2396766 | Frailty | Feeling hurt | A | A | 7 | 114318071 | <0.001 | 35.296 |
| rs3959554 | Frailty | Feeling hurt | G | G | 15 | 41443924 | 0.004 | 30.453 |
| rs4146140 | Frailty | Feeling hurt | T | T | 10 | 61885362 | <0.001 | 32.411 |
| rs4952693 | Frailty | Feeling hurt | T | T | 2 | 44151808 | <0.001 | 30.865 |
| rs56299474 | Frailty | Feeling hurt | A | A | 8 | 21992804 | <0.001 | 29.166 |
| rs583514 | Frailty | Feeling hurt | C | C | 3 | 173114167 | <0.001 | 34.685 |
| rs8089807 | Frailty | Feeling hurt | T | T | 18 | 39322639 | 0.002 | 32.721 |
| rs82334 | Frailty | Feeling hurt | C | C | 4 | 3225371 | <0.001 | 37.785 |
| rs9275160 | Frailty | Feeling hurt | A | A | 6 | 32652620 | <0.001 | 114.781 |
| rs12739243 | Frailty | Feeling tense | C | C | 1 | 210302043 | <0.001 | 35.295 |
| rs1363103 | Frailty | Feeling tense | C | C | 5 | 103917837 | 0.001 | 30.126 |
| rs17612102 | Frailty | Feeling tense | C | C | 15 | 52264094 | <0.001 | 29.575 |
| rs2071207 | Frailty | Feeling tense | C | C | 3 | 50159844 | 0.001 | 30.583 |
| rs3959554 | Frailty | Feeling tense | G | G | 15 | 41443924 | <0.001 | 30.453 |
| rs4146140 | Frailty | Feeling tense | T | T | 10 | 61885362 | <0.001 | 32.411 |
| rs4952693 | Frailty | Feeling tense | T | T | 2 | 44151808 | <0.001 | 30.865 |
| rs56299474 | Frailty | Feeling tense | A | A | 8 | 21992804 | <0.001 | 29.166 |
| rs583514 | Frailty | Feeling tense | C | C | 3 | 173114167 | <0.001 | 34.685 |
| rs8089807 | Frailty | Feeling tense | T | T | 18 | 39322639 | 0.005 | 32.721 |
| rs82334 | Frailty | Feeling tense | C | C | 4 | 3225371 | <0.001 | 37.785 |
| rs9275160 | Frailty | Feeling tense | A | A | 6 | 32652620 | <0.001 | 114.781 |
